# Supplementary material for: Fluorogenic CRISPR for genomic DNA imaging
Source: Nat Commun. 2024 Jan 31;15:934. doi: 10.1038/s41467-024-45163-9 (PMC10831063; doi:10.1038/s41467-024-45163-9)
Supplement: Supplementary file 1 — Supplementary Information [file 41467_2024_45163_MOESM1_ESM.pdf]

SUPPLEMENTARY INFORMATION

Fluorogenic CRISPR for genomic DNA imaging

Zhongxuan Zhang[1,2,3,4#], Xiaoxiao Rong[1,5#], Tianjin Xie[1,6], Zehao Li[1,5], Haozhi Song[1], Shujun Zhen[6], Haifeng Wang[7], Jiahui Wu[8], Samie R. Jaffrey[9], Xing Li[1, 2, 3, 4*]

[1] Beijing Institute of Life Sciences, Chinese Academy of Science, Beijing, 100101, China

[2] Department of Respiratory and Critical Care Medicine, The Affiliated Hospital of Southwest Medical University, Luzhou, 646000, Sichuan, China

[3] University of Chinese Academy of Sciences, Beijing, 100049, China

[4] State Key Laboratory of Integrated Management of Pest Insects and Rodents, Institute of Zoology, Chinese Academy of Sciences, Beijing 100101, China

[5] College of Life Science, Hebei University, Baoding, Hebei, 071002, China

[6] School of Chemistry and Chemical Engineering, Southwest University, Beibei District, Chongqing, 400715, China

[7] School of Life Sciences, Tsinghua-Peking Center for Life Sciences, Center for Synthetic and Systems Biology, Tsinghua University, Beijing 100084, China

[8] Department of Chemistry, University of Massachusetts, Amherst, MA 01003, USA

[9] Department of Pharmacology, Weill Cornell Medicine, Cornell University, New York, NY 10065, USA

[#]These two authors contributed equally to the work.
*Corresponding author. E-mail: lix@biols.ac.cn

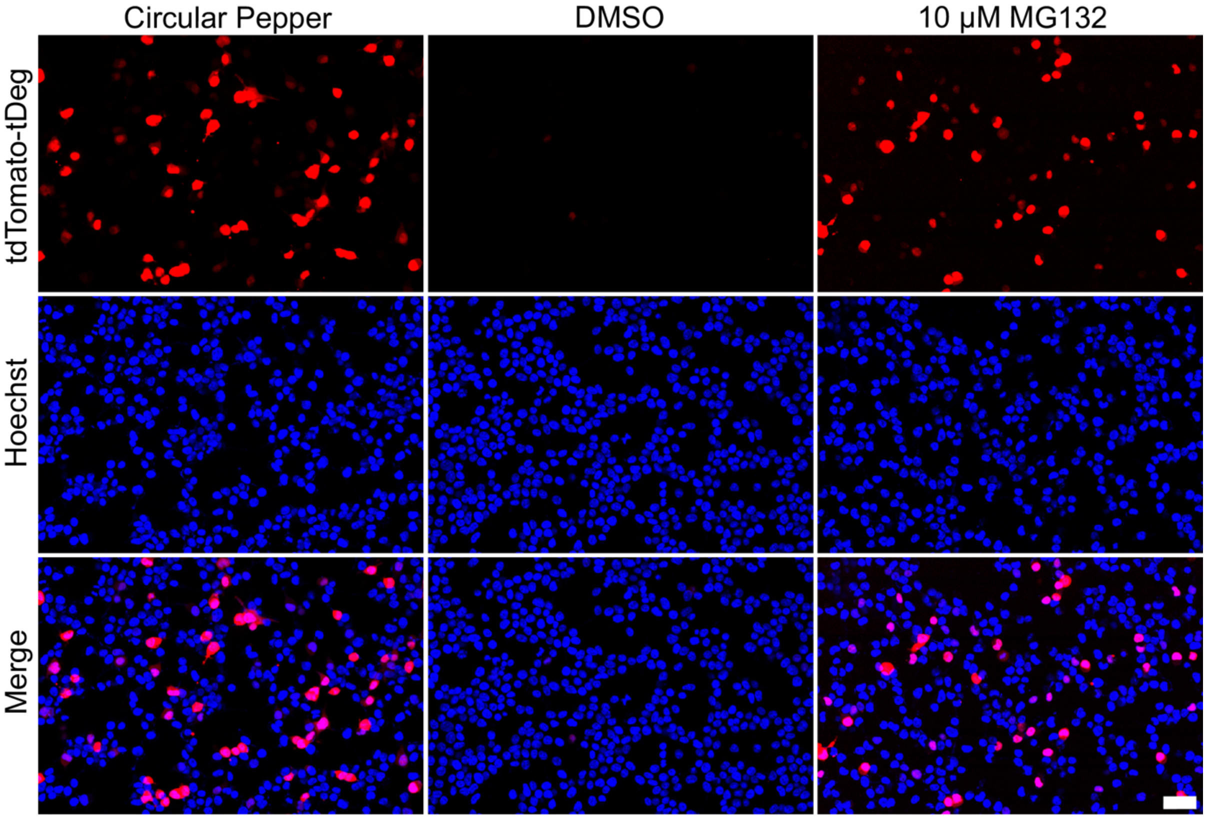

Supplementary Figure 1. Pepper prevents the proteasomal degradation of tDeg-fused fluorescent proteins in cells.

To test if tDeg-fused fluorescent proteins are degraded in cells and examine whether Pepper can prevent degradation, we transfected plasmids that express tdTomato-tDeg and circular Pepper in HEK293T cells. When cells expressed tdTomato-tDeg reporter only (treated with vehicle DMSO), minimal red fluorescence was detected (middle column). However, when tdTomato-tDeg was co-expressed with circular Pepper, the red fluorescence of tdTomato-tDeg was restored (left column).

To further validate whether tDeg causes protein instability by proteasomal degradation, cells were then treated with a proteasome inhibitor (10 μM MG132) for 7 h[1]. When proteasome activity was inhibited by treatment of MG132 (right column), the red fluorescence of tdTomato-tDeg was restored.

Thus, these data confirmed that the tDeg tag markedly reduces the stability of tdTomato by inducing proteasomal degradation. In addition, Pepper can prevent proteasomal degradation of a tDeg-fused fluorescent protein, which is consistent with previous results[1]. All cells were stained with Hoechst dye (1.0 μg/ml). Scale bar, 50 μm.

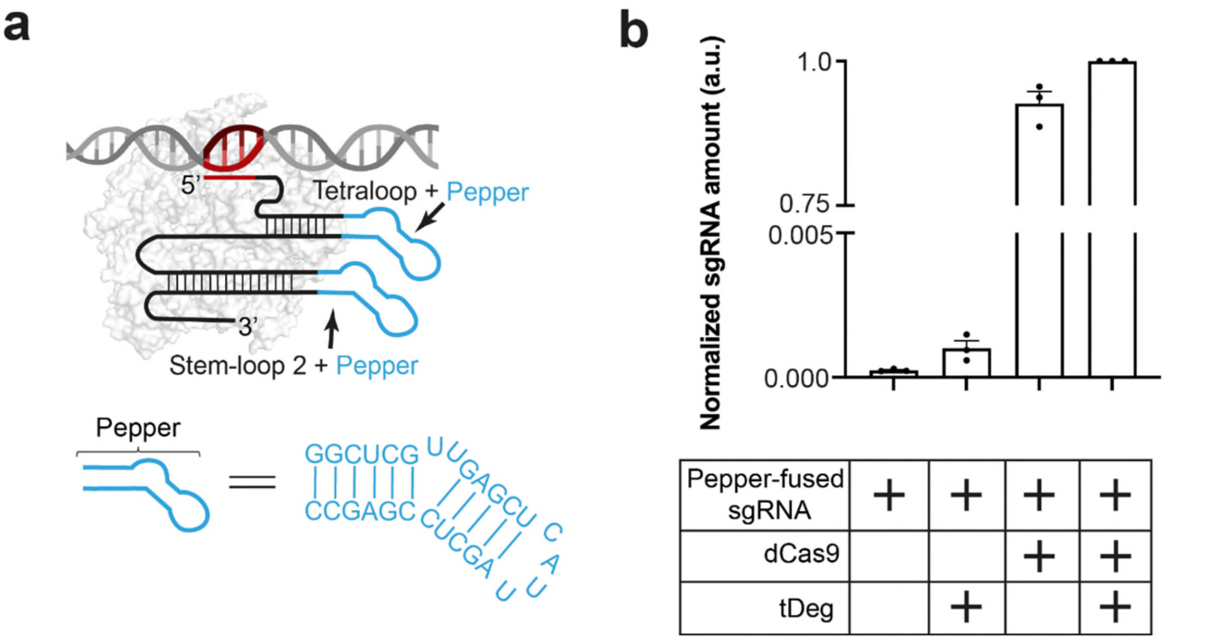

Supplementary Figure 2. Real-time quantitative PCR (RT-qPCR) verification of Pepper-fused sgRNA level in complex with tDeg and dCas9 protein.

a, Schematic of Pepper-fused sgRNA with dCas9. According to the crystal structure of Cas9-sgRNA complex[2], the tetraloop and stem-loop 2 in sgRNA do not make any contacts with Cas9. In addition, substitutions and deletions in the tetraloop and stem loop 2 regions of the sgRNA sequence do not affect Cas9 catalytic function[3-5]. Therefore, Pepper (blue) are inserted at tetraloop and stem-loop 2 regions, respectively. Cas9-binding region (black), and genomic DNA target region (spacer, red) of sgRNA are shown. The sequence of Pepper is shown below. The sequence of spacer and PAM are shown in Supplementary Table 2. Supplementary Fig. 2a was created with BioRender.com.

b, RT-qPCR analysis of Pepper-fused sgRNAs levels in different conditions. We sought to test whether Pepper-fused sgRNA will be stabilized without dCas9 or tDeg-fused fluorescent protein in cellular environment. To do this, we measured expression level of Pepper-fused sgRNA levels in the presence and absence of dCas9 and/or tDeg by RT-qPCR. Source data are provided as a Source Data file.

When only expressed Pepper-fused sgRNA or co-expressed with tDeg, the RNA level was extremely low. These data demonstrate that Pepper-fused sgRNA is highly unstable and cannot form stable complex with tDeg to induce background fluorescence. In contrast, when Pepper-fused sgRNAs were co-expressed with dCas9, the RNA level was highly increased. These data showed that Pepper-fused sgRNAs can form a stable complex with dCas9. In addition, the complex of Pepper-fused sgRNAs and dCas9 is able to recruit the tDeg-fusing fluorescent protein reporter for imaging targeted genomic loci.

All data are presented as the mean ± s.d.; n = 3 independent experiments; black dots represent individual data points; Pepper-fused sgRNA with dCas9 and tDeg group was set as the control group for each independent experiment.

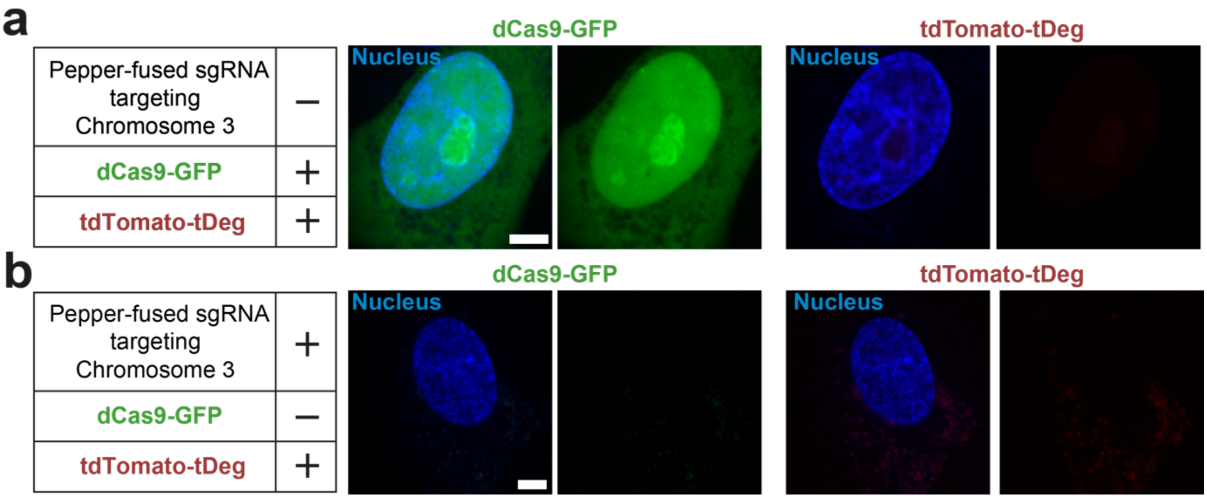

Supplementary Figure 3. fCRISPR could not image genomic loci lacking sgRNA or dCas9.

a, tDeg-fused fluorescent proteins did not readily image genomic loci when Pepper-fused sgRNAs were not expressed. We co-expressed dCas9-GFP and tdTomato-tDeg in living U2OS cells. In the absence of Pepper-fused sgRNAs, we could not observe the readily detectable puncta from both dCas9-GFP (left) and tdTomato-tDeg (right) reporter. In addition, dCas9-GFP exhibited a high background fluorescence with non-specific accumulation, whereas tdTomato-tDeg exhibited minimal background fluorescence.

b, Pepper-fused sgRNA were degraded in the absence of dCas9. U2OS cells were co-expressed with Pepper-fused sgRNA and tdTomato-tDeg. Without dCas9, Pepper-fused sgRNAs could not form dCas9-sgRNA complex, then were degraded by RNase[5] (Supplementary Fig. 2b). tdTomato-tDeg reporter were destroyed in the absence of dCas9-sgRNA complex formation. Therefore, we could not observe the readily detectable puncta from tdTomato-tDeg reporter (right). These results suggest that both Pepper-fused sgRNA and tdTomato-tDeg were degraded in the absence of dCas9. Together, fCRISPR could not image genomic loci lacking dCas9. These imaging data are consistent with qPCR data (Supplementary Fig. 2b)

All cells were stained with Hoechst dye (1.0 µg/ml). 50 cells were analyzed in each figure with similar results. Scale bar, 5 µm.

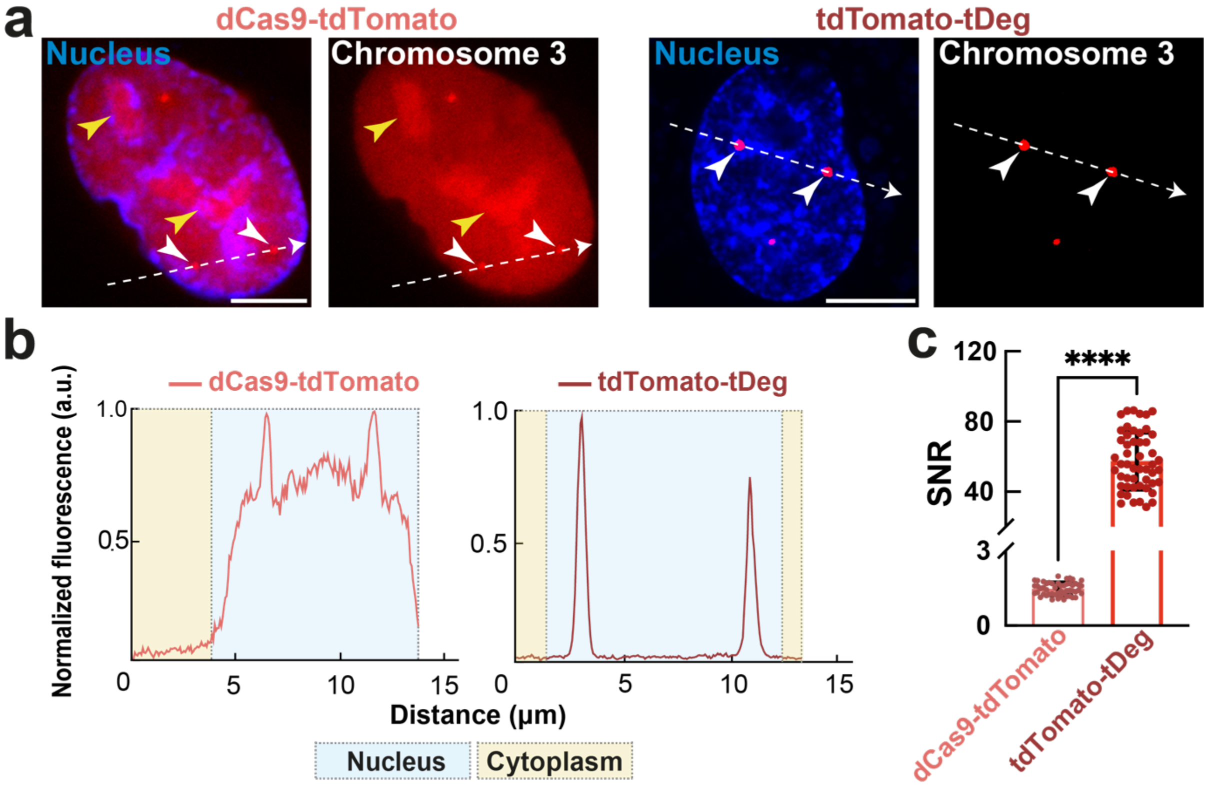

Supplementary Figure 4. fCRISPR with tdTomato-tDeg shows high SNR over the conventional CRISPR with dCas9-tdTomato for genomic loci imaging.

a. Comparison of genomic loci labeling (white arrowheads) between conventional CRISPR and fCRISPR with the identical tdTomato reporter. We compared the fluorogenic ability between conventional CRISPR with dCas9-tdTomato and fCRISPR with tdTomato-tDeg reporters. To do this, we expressed these two systems targeting Chromosome 3 repetitive loci in U2OS cells, respectively. The imaging results showed the background fluorescence of tdTomato-tDeg reporter in the nucleus is significantly lower than dCas9-tdTomato reporter. In addition, conventional CRISPR with dCas9-tdTomato reporter produces nonspecific accumulation of fluorescence (yellow arrowheads). In contrast, fCRISPR with tdTomato-tDeg reporter did not display background fluorescence in the nucleolus. The white dotted lines run through the cytoplasm to the nucleus to produce the plot profile in Supplementary Fig. 4b. All cells were stained with Hoechst dye (1.0 μg/ml). Scale bar, 5 μm.

b. Fluorescence profiles of labeled Chromosome 3 loci with dCas9-tdTomato reporter (peach) and tdTomato-tDeg reporter (red). Background fluorescence of dCas9-tdTomato (peach lines) in the nucleus (light blue area) is higher than that of tdTomato-tDeg (red lines). Fluorescence plots were generated from the white dotted lines in Supplementary Fig. 4a. Source data are provided as a Source Data file.

c. fCRISPR with tdTomato-tDeg (red, right) shows higher SNR compared to the CRISPR with dCas9-tdTomato (peach, left). Data are represented as means ± standard deviation for tdTomato-tDeg (1.470 ± 0.2557) and dCas9-tdTomato (57.48 ± 16.24). $p<0.0001$ by Wilcoxon test; 22 cells (51 fluorescent puncta) were analyzed. Source data are provided as a Source Data file.

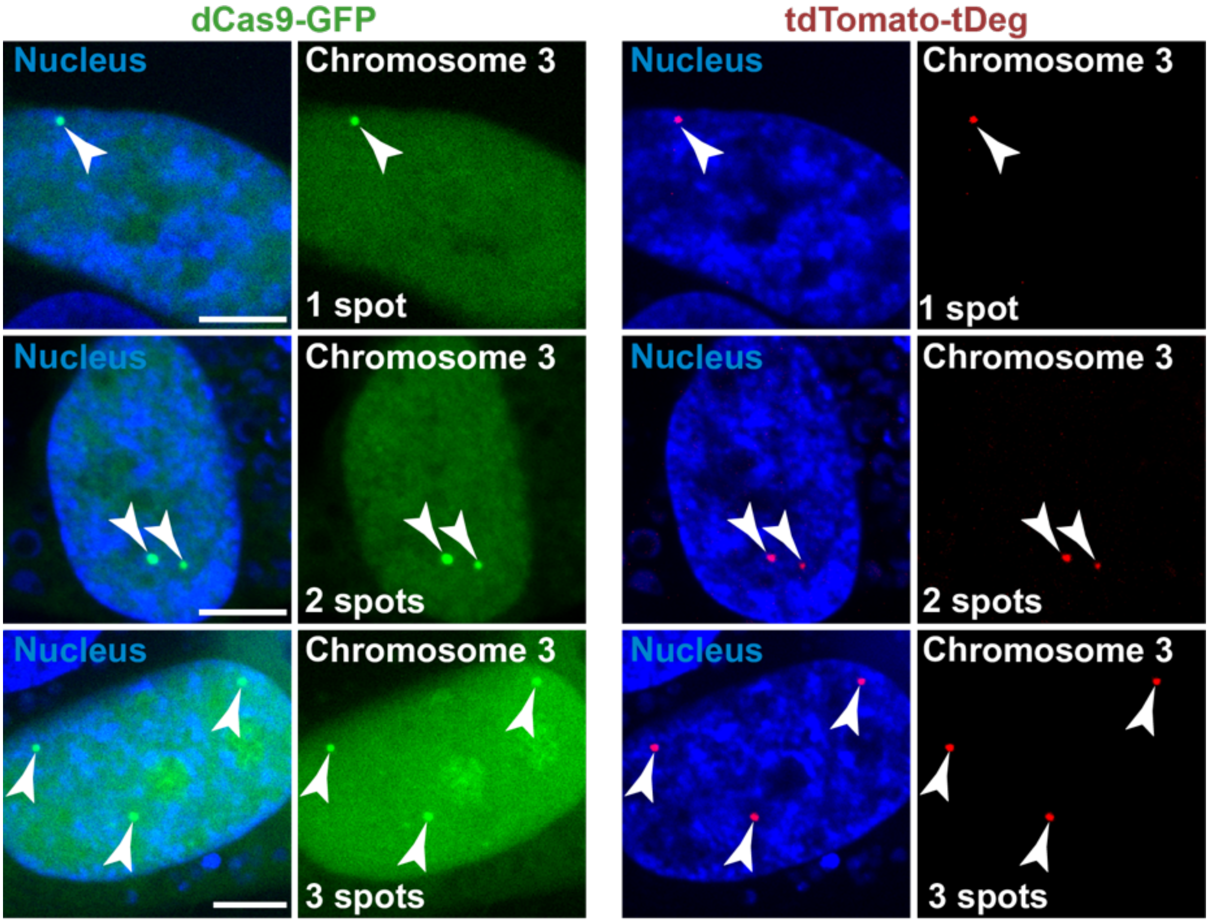

Supplementary Figure 5. fCRISPR is able to image one to three Chromosome 3 loci numbers in various U2OS cells.

fCRISPR labeling of Chromosome 3 loci showed one to three fluorescent puncta in U2OS cells. Unlike normal diploid cells, the U2OS cell karyotype is highly abnormal[6]. With fCRISPR, we readily observed the different numbers (1-3) of Chromosome 3 loci (white arrowheads) in U2OS cells, which matched the results from previous reports[5]. All cells were stained with Hoechst dye (1.0 µg/ml). Scale bar, 5 µm.

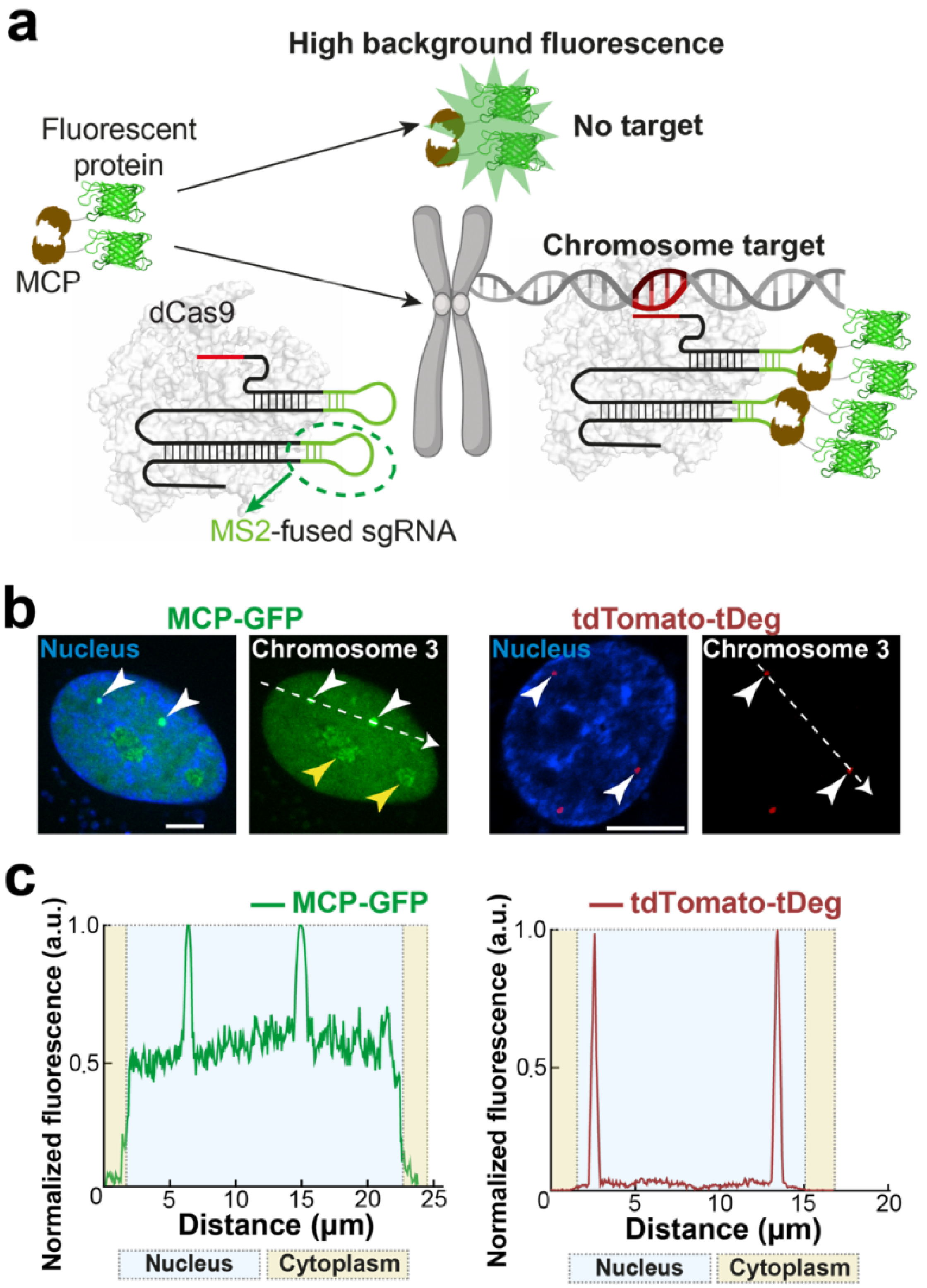

Supplementary Figure 6. Comparison of genomic loci imaging between MCP-fused fluorescent protein and fluorogenic protein.

a, Schematic of CRISPR with MS2-recruiting MCP-fused fluorescent protein for genomic loci imaging. In contrast, fCRISPR using a tDeg-fused fluorescent protein, which is degraded in the cell, MCP (MS2 coat protein, brown)-fused fluorescent protein (green) is stable, leading to background fluorescence. When MCP-fused fluorescent

protein binds to MS2 (green line)-fused sgRNA and dCas9 (blue), genomic loci can be targeted and fluorescent puncta can be observed[4]. Supplementary Fig. 6a was created with BioRender.com.

b-c, Comparison of genomic loci labeling (white arrows) between CRISPR with MCP-GFP reporter (left) and fCRISPR with tdTomato-tDeg reporter (right). To image genomic loci using CRISPR with MCP-GFP reporter, U2OS cells were transfected with the MS2-fusing sgRNA targeting Chromosome 3, dCas9-mCherry, and MCP-GFP. We found MCP-GFP labeled fluorescent puncta (left, white arrowheads), nonspecific fluorescent protein accumulation (yellow arrowheads), and high background fluorescence in the nucleus. In contrast, the background fluorescence of tdTomato-tDeg reporter (right) in the nucleus and cytoplasm is significantly lower than MCP-GFP reporter (left). Therefore, CRISPR with MCP-GFP reporters exhibits strong fluorescent background and nonspecific accumulation of fluorescent protein in the nucleolus. Source data are provided as a Source Data file.

The white dotted lines produce the plot profile in Supplementary Fig. 6c. All cells were stained with Hoechst dye (1.0 µg/ml). Scale bar, 5 µm.

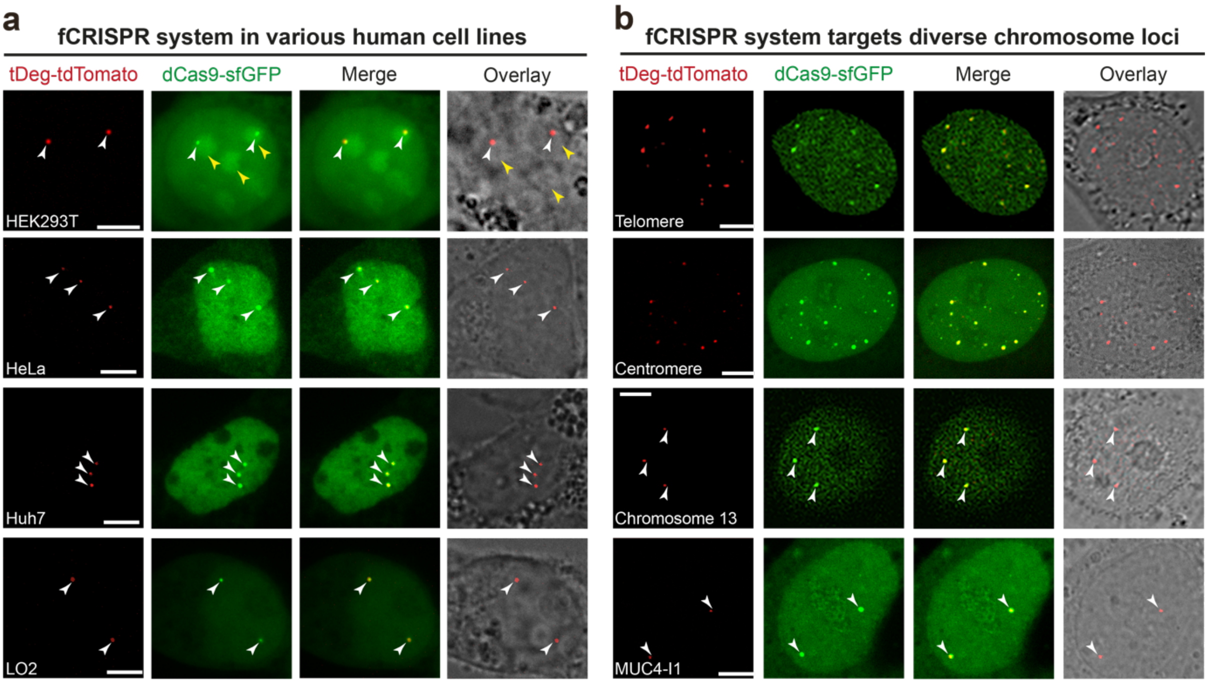

Supplementary Figure 7. Verification of fCRISPR in various high-copy (>100 copies) genome loci tracking and different human cell lines.

a, fCRISPR enables genomic loci imaging in various living human cell lines. To test if fCRISPR is capable of imaging Chromosome 3 (~500 copies) in other living human cell lines, we transfected fCRISPR with tdTomato-tDeg reporter in HEK293T, HeLa, Huh7, and LO2 cell lines, respectively. In all cell types observed, red fluorescent puncta from tdTomato-tDeg reporters were readily detected only when expressed using fCRISPR. These red fluorescent puncta colocalized with green fluorescent puncta from dCas9-GFP reporter in cells.

b, fCRISPR is able to image different genomic loci. To test if fCRISPR is able to image genomic loci in different chromosomes, we transfected fCRISPR with tdTomato-tDeg reporter targeting telomeres (>500 copies), centromere (>500 copies), Chromosome 13 (~500 copies), and *MUC4*-Intron 1 (*MUC4*-I1, ~90 copies), respectively, in U2OS cells. In all genomic loci observed, red fluorescent puncta from tdTomato-tDeg reporter were readily detected only when expressed using fCRISPR. These red fluorescent puncta colocalized with green fluorescent puncta from dCas9-GFP reporter in cells.

a and **b**, The signal spots of dCas9-GFP and bright field were shown. To verify the labeling efficiency of fCRISPR system, we used dCas9-GFP for validation. We observed the co-labeling signal spots (white arrowheads) of tdTomato-tDeg and dCas9-GFP in the same cell. Nucleolar dCas9-GFP accumulation (yellow arrowheads) was shown. Scale bar, 5 μm. These experiments were performed three times with similar results.

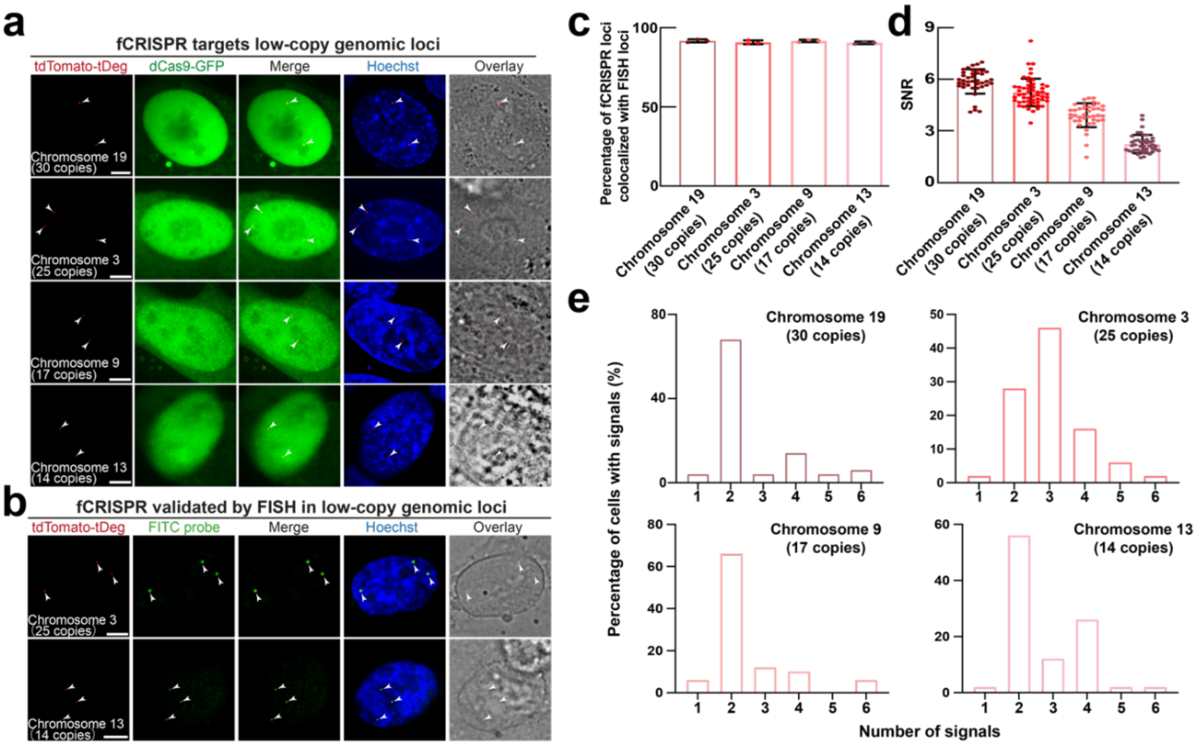

Supplementary Figure 8. Verification of fCRISPR for low-copy genome loci tracking.

a, fCRISPR is able to image different low-copy genomic loci in living U2OS cells. Representative images of fCRISPR and conventional CRISPR in low-copy genomic loci imaging. To test if fCRISPR is able to image low-copy genomic loci in different chromosomes, we transfected fCRISPR with tdTomato-tDeg reporter targeting low-copy genomic loci on Chromosome 19 (30 copies), Chromosome 3 (25 copies), Chromosome 9 (17 copies), and Chromosome 13 (14 copies), respectively, in U2OS cells. In all genomic loci observed, red fluorescent puncta from tdTomato-tDeg reporter were readily detected only when expressed using fCRISPR (white arrowheads). However, conventional CRISPR with dCas9-GFP reporter cannot observe signals at all low-copy genomic loci[7,8]. Scale bar, 5 μm. These experiments were performed at least three times with similar results.

b, Representative images of fCRISPR labeled low-copy genomic loci in fixed cells with FISH validation. We expressed fCRISPR imaging system targeted various low-copy genomic loci in U2OS cells and subsequently fixed the cells to incubate FITC-fused DNA FISH probes. After hybridization, images were acquired with the confocal microscopy. Representative imaging of Chromosome 3 (25 copies, top) and Chromosome 13 (14 copies, bottom) were shown respectively. We observed the co-labeling signal spots (white arrowheads) of tdTomato-tDeg (red) and FITC-fused probes (green) in the same cell. Scale bar, 5 μm. These experiments were performed at least three times with similar results.

c, Quantification of the specificity when imaging low-copy genome loci using fCRISPR in (**b**). The specificity of fCRISPR in low-copy genomic loci is around 90-92%. Values are means ± s.d.. n= 150 cells per condition. Source data are provided as a Source Data file.

d, Quantification of the sensitivity when imaging low-copy genome loci using fCRISPR. The SNR of these fCRISPR labeled low-copy genomic loci is between 2.2-5.8. Values are means ± s.d.. n= 22 cells per condition. Source data are provided as a Source Data file.

e, Quantification of the number of signals when imaging low-copy genome loci using fCRISPR. The number of signals is variable in U2OS cells, ~60% of cells showed 2 signals when imaged low-copy numbers in Chromosome 9, 13, and 19, while 46% of cells showed 3 signals in Chromosome 3. Values are means ± s.d.. n= 50 cells per condition. Source data are provided as a Source Data file.

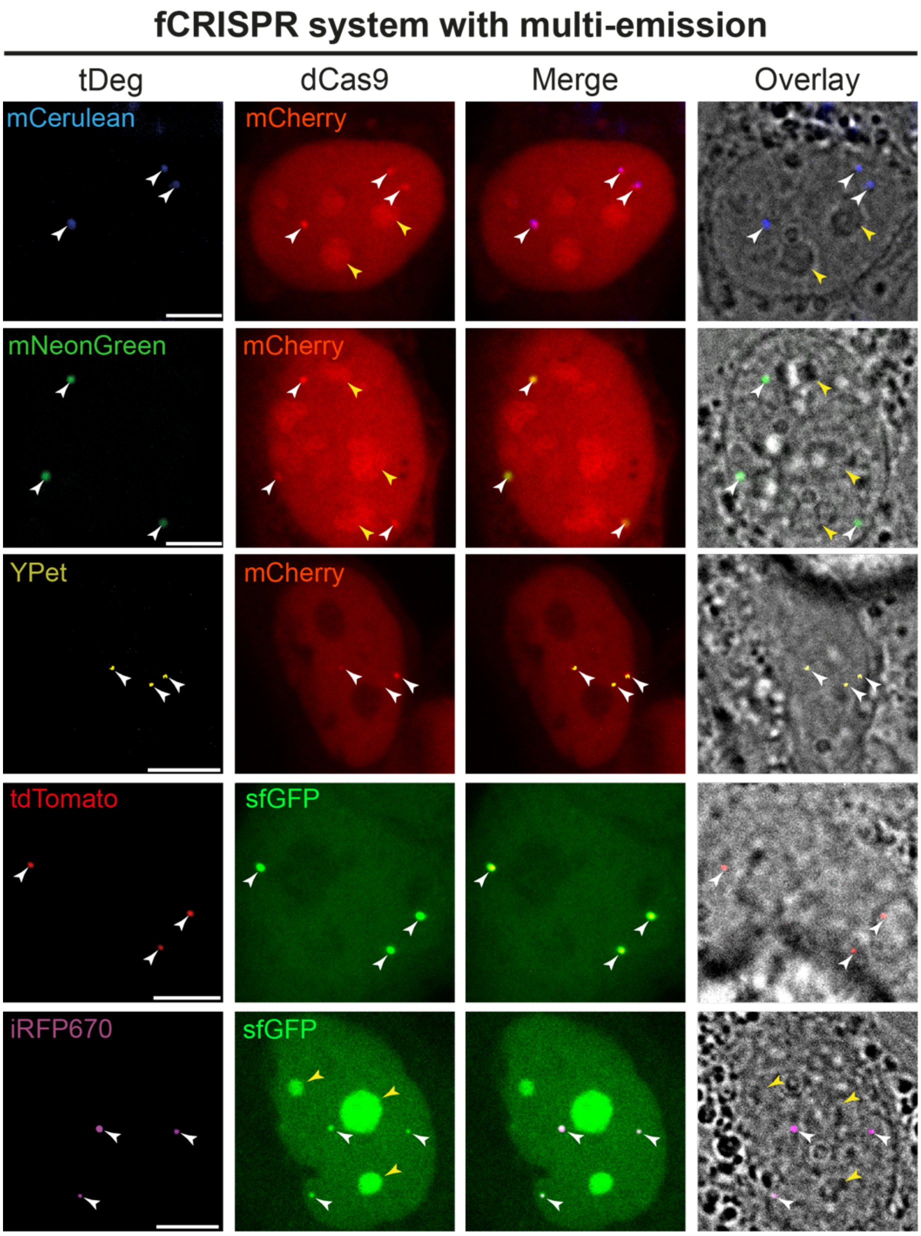

Supplementary Figure 9. Verification of multi-color genomic loci imaging.

fCRISPR enables multi-color imaging by modulating tDeg-fused fluorescent proteins. The dCas9 signal spots and bright fields were displayed. We utilized dCas9-mCherry to validate the mCerulean-tDeg, mNeonGreen-tDeg, and YPet-tDeg. dCas9-GFP was validated using tdTomato-tDeg and iRFP670-tDeg to prevent the overlap of excitation and emission spectra. Co-labeling signal spots (white arrowheads) of tDeg and dCas9 were observed in the same cell. Nucleolar dCas9-GFP accumulation (yellow arrowheads) was shown. Scale bar, 5 μm.

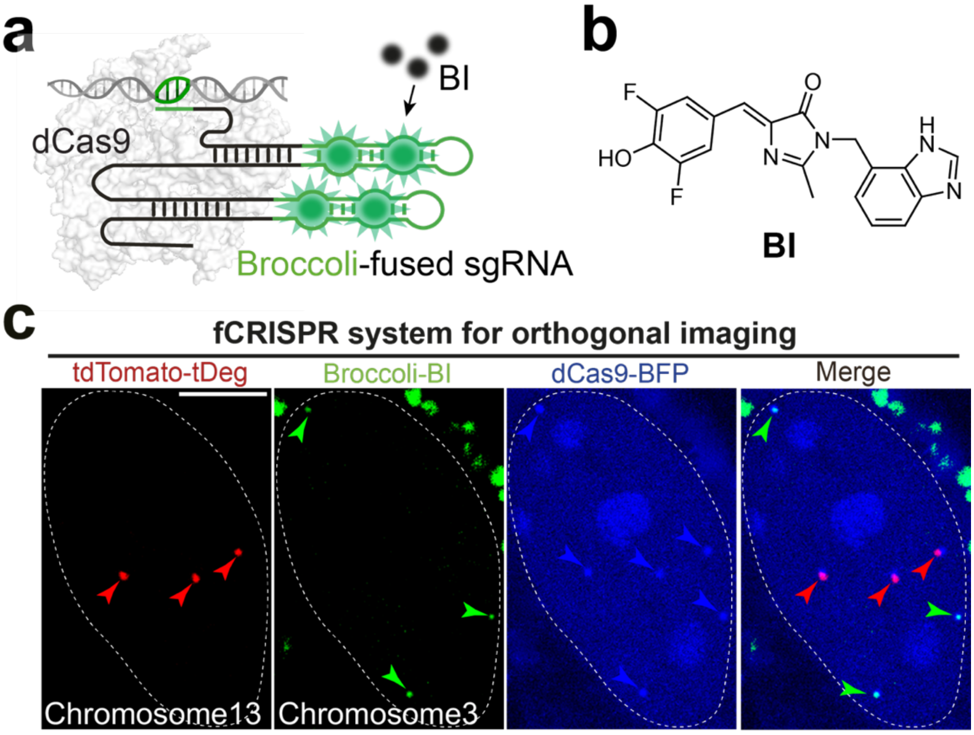

Supplementary Figure 10. The Broccoli-fused CRISPR for orthogonal genomic loci imaging with fCRISPR.

a, Schematic of Broccoli-fused CRISPR with small-molecule BI fluorophore for genomic loci imaging. Unlike Pepper, which induces the fluorescence of fluorogenic proteins, Broccoli binds and induces the fluorescence of an otherwise nonfluorescent BI dye[9]. Broccoli-fused sgRNA is designed by inserting two dimeric Broccoli RNA aptamer (green line) into the tetraloop and stem-loop2 of sgRNA[5,9]. Supplementary Fig. 10a was created with BioRender.com.

b, Chemical structures of BI. Broccoli binds BI to produce bright green fluorescence (Ex = 470 nm, Em = 505 nm)[9].

c, Orthogonal live-cell imaging of high-copy genomic loci Chromosome 3 (~500 copies) and Chromosome 13 (~500 copies) in U2OS cells. We co-expressed fCRISPR using tdTomato-tDeg reporter, and Broccoli-fused CRISPR with BI reporter. Furthermore, we utilized dCas9-BFP (blue arrowheads) to validate the targeted loci. Both red and green fluorescent puncta were readily detected in the presence of BI dyes (10 μM) and colocalized with the blue dCas9-BFP reporter in the merged image. These results demonstrate that fCRISPR can function as an orthogonal live-cell imaging platform together with other CRISPR imaging techniques. Although the sgRNAs with Broccoli exhibit fluorogenic ability towards BI dyes in the nucleus, the exogenously added BI dyes exhibit nonspecific background on their own This issue is widespread for other fluorogenic aptamers with exogenously added fluorophores. This may limit the utility of this system. Nevertheless, these data show that fCRISPR can be used for multiplexed imaging of different genomic loci when coupled with other CRISPR-based imaging systems. Scale bar, 5 μm.

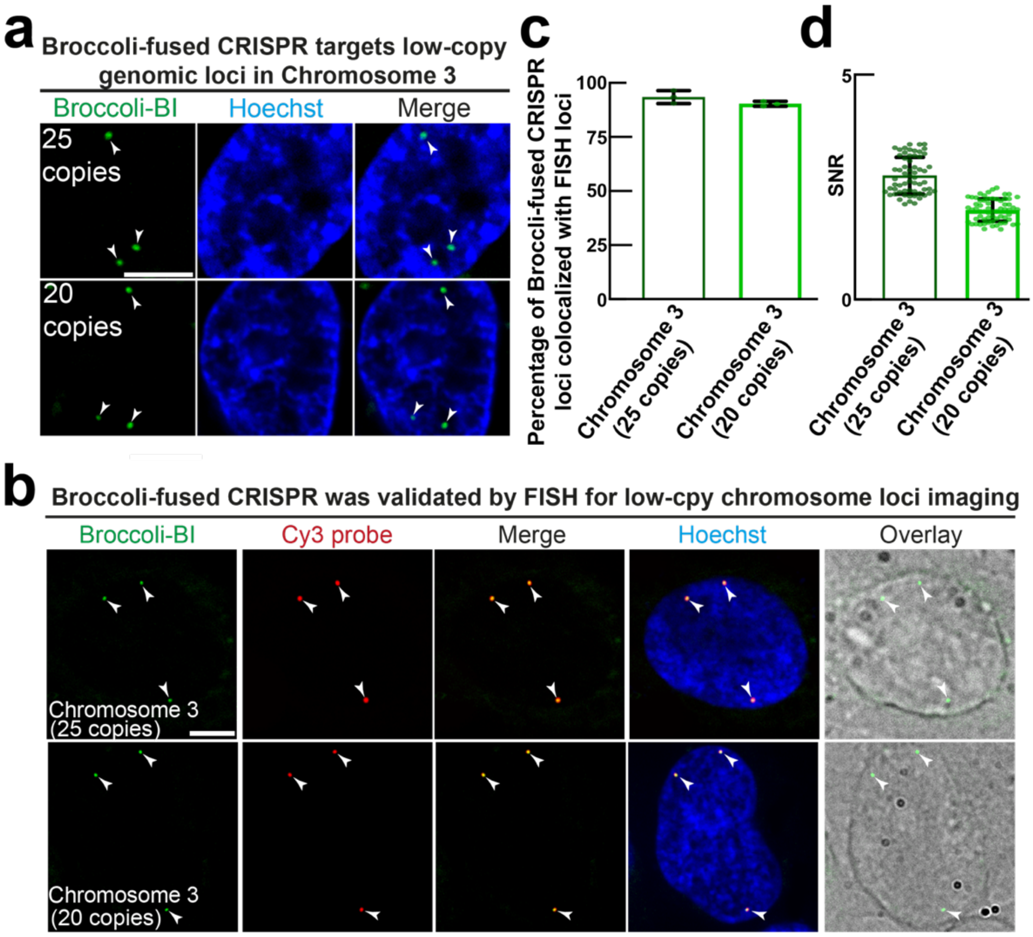

Supplementary Figure 11. Verification of Broccoli-fused CRISPR for various low-copy genome loci tracking.

a, Broccoli-fused CRISPR system enables low-copy genomic loci imaging in living U2OS cells. We co-expressed dCas9 and Broccoli-fused sgRNA targeting low-copy genomic loci with 25 copies (top) or 20 copies (bottom) in Chromosome 3. Images were acquired with confocal microscopy after incubation with BI dye (10 μM) and Hoechst dye (1.0 μg/ml) for 1 h. We observed three fluorescent puncta in both U2OS cells (white arrowheads). Scale bar, 5 μm. These experiments were performed three times with similar results.

b, FISH validation demonstrated that Broccoli-fused CRISPR can image low-copy genomic loci in Chromosome 3. We expressed Broccoli-fused CRISPR in U2OS cells and subsequently fixed the cells to incubate Cy3-fused DNA FISH probes and BI dye (10 μM). After FISH hybridization, we observed the co-labeling signal spots (white arrowheads) of Broccoli-fused CRISPR with BI reporter and Cy3-fused FISH probes in the same cell. Representative imaging of 25 copies (top) and 20 copies (bottom) in Chromosome 3 were shown respectively. These experiments were performed three times with similar results. Scale bar, 5 μm.

c, Quantification of colocalization between FISH and Broccoli-fused CRISPR as in (**b**). The specificity of Broccoli-fused CRISPR in low-copy genomic loci is around 90-93%. Values are means ± s.d.. n= 120 cells per condition. Source data are provided as a Source Data file.

d, Quantification of SNR when imaging low-copy genome loci using Broccoli-fused CRISPR. The SNR of these Broccoli-fused CRISPR labeled low-copy genomic loci is between 2.0-2.8. Values are means ± s.d.. n= 20 cells per condition. Source data are provided as a Source Data file.

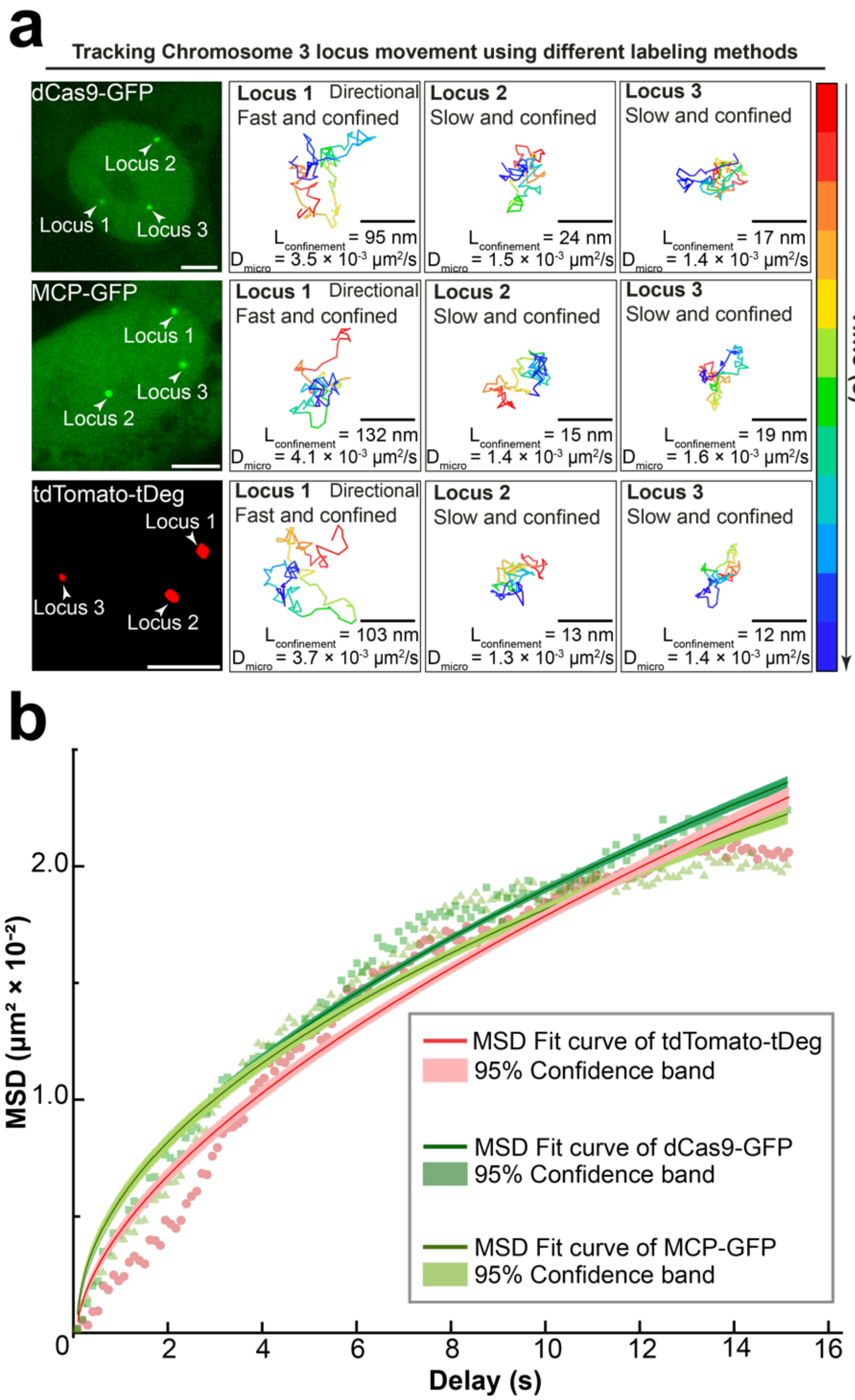

Supplementary Figure 12. Comparison between fCRISPR and two conventional CRISPR-based imaging systems for tracking chromosomal dynamics.

a, The single-particle trajectory tracking with fCRISPR and two conventional CRISPR[4,8] imaging systems. We compared the chromosomal heterogeneity using fCRISPR with two other conventional CRISPR systems, including CRISPR with dCas9-GFP reporter and MS2-fused CRISPR with MCP-GFP reporter. To do this, we performed fCRISPR and these two conventional CRISPR targeting Chromosome 3 in

U2OS cells. We observed three fluorescent puncta (white arrowheads). To determine the chromosomal dynamics heterogeneity, we analyzed the short-time scale (within 5s) confinement sizes ($L_{confinement}$) and microscopic diffusion coefficients (D_{micro}). One of the Chromosome 3 loci showed directional transport with longer displacements than the others, and all Chromosome 3 loci were movement-confined. By comparing the D_{micro} and $L_{confinement}$, the different CRISPR-based imaging systems showed the similar results. These results are consistent with fCRISPR with conventional CRISPR-based imaging systems in chromosomal dynamics analysis[7,10].

The time interval is 0.11 s, and the gradient colors of each trajectory represent the from 0 to 15 s. Scale bar for fluorescence figure, 5 μm. Scale bar for the trajectories, 0.2 μm. See Supplementary Movie 2-4. Source data are provided as a Source Data file.

b, The quantitative mean-squared displacement (MSD) curves of fCRISPR and two conventional imaging systems[4,8]. The MSD curves of fCRISPR with tdTomato-tDeg (red, D_{micro} = 1.85x10^{-3} μm^2/s), dCas9-GFP (dark green, D_{micro} = 2.2x10^{-3} μm^2/s), and MCP-GFP (light green, D_{micro} = 2.0x10^{-3} μm^2/s) were shown, and the colored shaded area represents the 95% fitting confidence interval. The MSD curves between fCRISPR and other two CRISPR-based systems had a similar trend, representing similar confinement characteristics. Therefore, fCRISPR is consistent with previous results and showed that the Chromosome 3 movements were highly confinement[7,10]. n= 45 cells per condition. Source data are provided as a Source Data file.

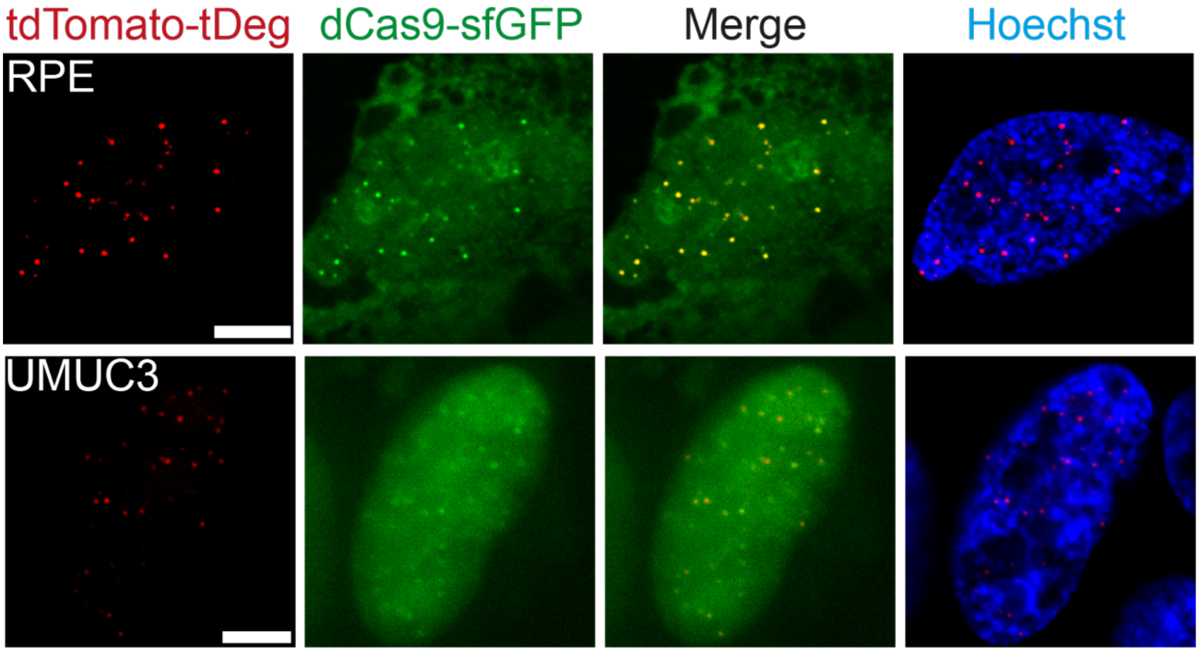

Supplementary Figure 13. Comparison of relative telomere length between fCRISPR with tdTomato-tDeg and CRISPR with dCas9-GFP in the same cell.

The representative images of labeled telomere fluorescence puncta in RPE and UMUC3 cells using fCRISPR with tdTomato-tDeg and CRISPR with dCas9-GFP. To detect the telomere length, we co-expressed dCas9-GFP, tdTomato-tDeg, Pepper-fused sgRNA targeting telomere in RPE (top) and UMUC3 (bottom) cells. The fluorescent reporters of tdTomato-tDeg reporter (red) and dCas9-GFP (green) reporter showed colocalization, validating the specificity of fCRISPR for imaging telomere. Also, the background fluorescence of tdTomato-tDeg reporter in the nucleus and cytoplasm is significantly lower than dCas9-GFP as shown. All cells were stained with Hoechst dye (1.0 μg/ml). Scale bar, 5 μm. These experiments were performed at least three times with similar results.

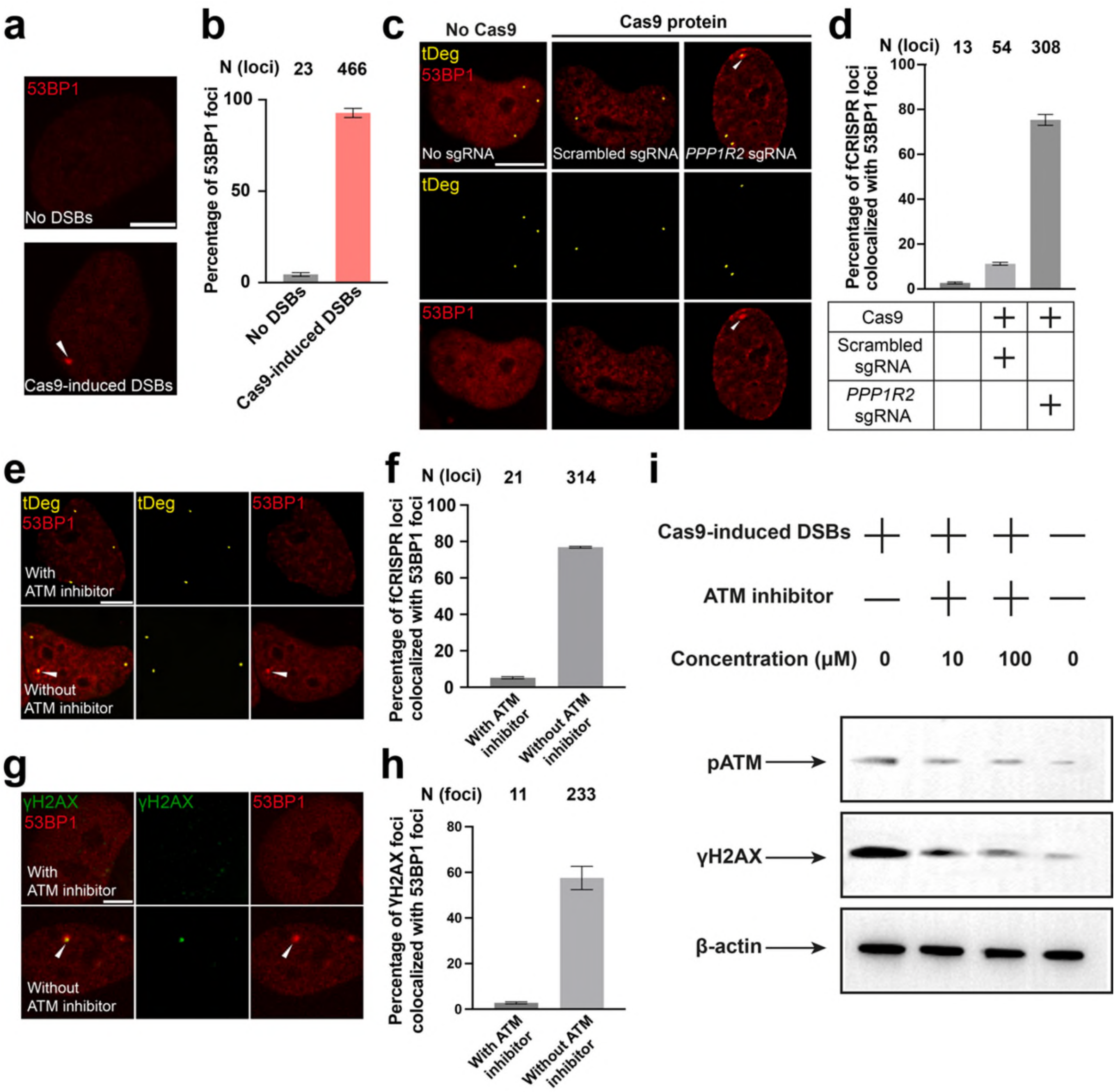

Supplementary Figure 14. 53BP1-Apple reporters can detect Cas9-induced DNA breaks and repairs.

a, Representative images of 53BP1-Apple expressing U2OS cells in the presence or absence of *PPP1R2*-editing CRISPR. We created a U2OS cell line to stably express 53BP1-Apple. To avoid the interference of pre-existing 53BP1-Apple foci caused by 53BP1-Apple high expression, we performed fluorescence-activated cell sorting to sort and collect cells with low 53BP1-Apple expression[11].

To determine if 53BP1-Apple foci is not pre-existing in U2OS cells without CRISPR-induced DSBs, we then transfected empty vector or *PPP1R2*-editing CRISPR in 53BP1-Apple expressing U2OS cells, respectively. We barely observed pre-existing 53BP1 foci in cells without *PPP1R2*-editing CRISPR transfection(top). In contrast, we observed an apparent 53BP1 foci (bottom, white arrowheads) after *PPP1R2*-editing CRISPR transfection. The foci likely represent the recruitment of 53BP1 at the Cas9-induced DSBs loci[11,12]. Scale bar, 5 μm. These experiments were performed at least three times with similar results.

b, Quantification of 53BP1-Apple foci in (**a**). Values are means ± s.d.. n= 500 cells per condition. Source data are provided as a Source Data file.

c, 53BP1-Apple foci are not colocalized to Chromosome 3 without expression of *PPP1R2*-editing CRISPR. To test the targeting specificity of Cas9-induced gene editing, we performed *PPP1R2*- or scrambled-targeted sgRNA with Cas9. We observed barely 53BP1 foci and fCRISPR loci colocalization in empty vector or scrambled-targeted sgRNA with Cas9 transfected cells. In contrast, fCRISPR loci and 53BP1-Apple foci (white arrowheads) were colocalized in the cells that expressed *PPP1R2*-editing CRISPR. These results further clarified the recruitment of 53BP1 at the Cas9-induced DNA break site[11,12]. Scale bar, 5 μm. These experiments were performed at least three times with similar results.

d, Quantification of colocalization between fCRISPR loci and 53BP1-Apple foci in (**c**). Values are means ± s.d.. n= 400 cells per condition. Source data are provided as a Source Data file.

e, Representative live-cell images showing inhibition of 53BP1-Apple recruitment by ATM inhibitor. We used an ATM inhibitor KU-0055933, a commonly used inhibitor to block ATM-dependent phosphorylation[13,14], to hinder the 53BP1-Apple recruitment to the DSBs locus. We added KU-0055933 (100 μM) in 53BP1-Apple stably expressing U2OS cells that expressed *PPP1R2*-targeted sgRNA with Cas9 and fCRISPR. After adding KU-0055933 for 1 h, we observed barely 53BP1 foci existed. Without adding KU-0055933, we observed fCRISPR loci colocalized with 53BP1 foci. These results demonstrate ATM activity is required for 53BP1 recruitment to DSBs loci. Scale bar, 5 μm. These experiments were performed at least three times with similar results.

f, Quantification of colocalization between fCRISPR loci and 53BP1-Apple foci incubated with or without ATM inhibitor KU-0055933 in (**e**). Values are means ± s.d.. n= 400 cells per condition. Source data are provided as a Source Data file.

g, Representative immunofluorescence images showing the disappearance of 53BP1-Apple and γH2AX foci with ATM inhibition. We next asked whether truncated 53BP1, similar to classic biomarker γH2AX, undergoes phosphorylation following DNA DSBs. Phosphorylated histone H2AX (γH2AX) functions in the recruitment of DNA damage response proteins to DSBs. ATM also colocalizes with γH2AX at DSBs sites following its auto-phosphorylation. Thus, we tried to observe the localization between 53BP1 and γH2AX upon the addition of an ATM inhibitor in the same cell. To do this, we transfected *PPP1R2*-targeted sgRNA with Cas9 in 53BP1-Apple expressing U2OS cells and added KU-0055933 (100 μM). After adding KU-0055933 for 1 h, we fixed the cells and labeled γH2AX with Alexa Fluor 488 for immunofluorescence. We observed both truncated form of 53BP1 and γH2AX formation were abrogated when ATM was suppressed. These results indicate that ATM activity is required for the recruitment of both 53BP1-Apple and γH2AX. Scale bar, 5 μm. These experiments were performed at least three times with similar results.

h, Quantification of colocalization between γH2AX loci and 53BP1-Apple foci incubated with or without ATM inhibitor KU-0055933 in (**g**). Values are means ± s.d.. n= 400 cells per condition. Source data are provided as a Source Data file.

i, Western blots validate phosphorylation levels for DSBs-responsive endogenous proteins by adding ATM inhibitors. The data shown here is a representative image from 3 independent cell cultures. The whole cropped blot is shown in Supplementary Fig. 20.

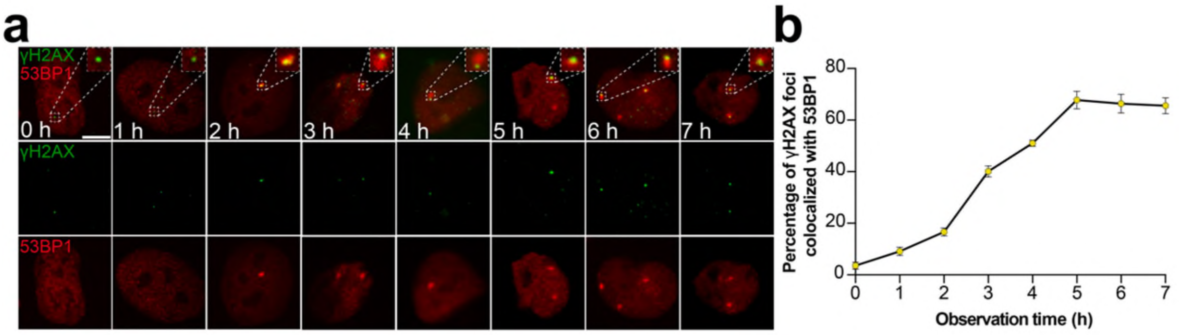

Supplementary Figure 15. 53BP1-Apple reporter in U2OS cells localization to Cas9-induced DSBs sites in H2AX

Representative immunofluorescence shows the colocalization between γH2AX and 53BP1 foci after the transfection of *PPP1R2*-targeted sgRNA with Cas9. γH2AX foci appearance can signal the DNA breaks[15-17]. After 5h transfection (the observation time at 0 h in the top-left figure), we then fixed 53BP1-Apple stably expressing U2OS cells and labeled γH2AX with Alexa Fluor 488 for immunofluorescence at different time points (**a**). We observed that the γH2AX and 53BP1 foci colocalization increased from 0 h (~3.5% of 200 cells, 5h transfection) to 7 h (~67.7% of 200 cells, 12 h transfection). These data demonstrated that 53BP1-Apple foci were colocalized with γH2AX foci and likely signal Cas9-induced DNA breaks. Scale bar, 5 µm. n=200 cells for each time point (**b**). These experiments were performed at least three times with similar results. Source data are provided as a Source Data file.

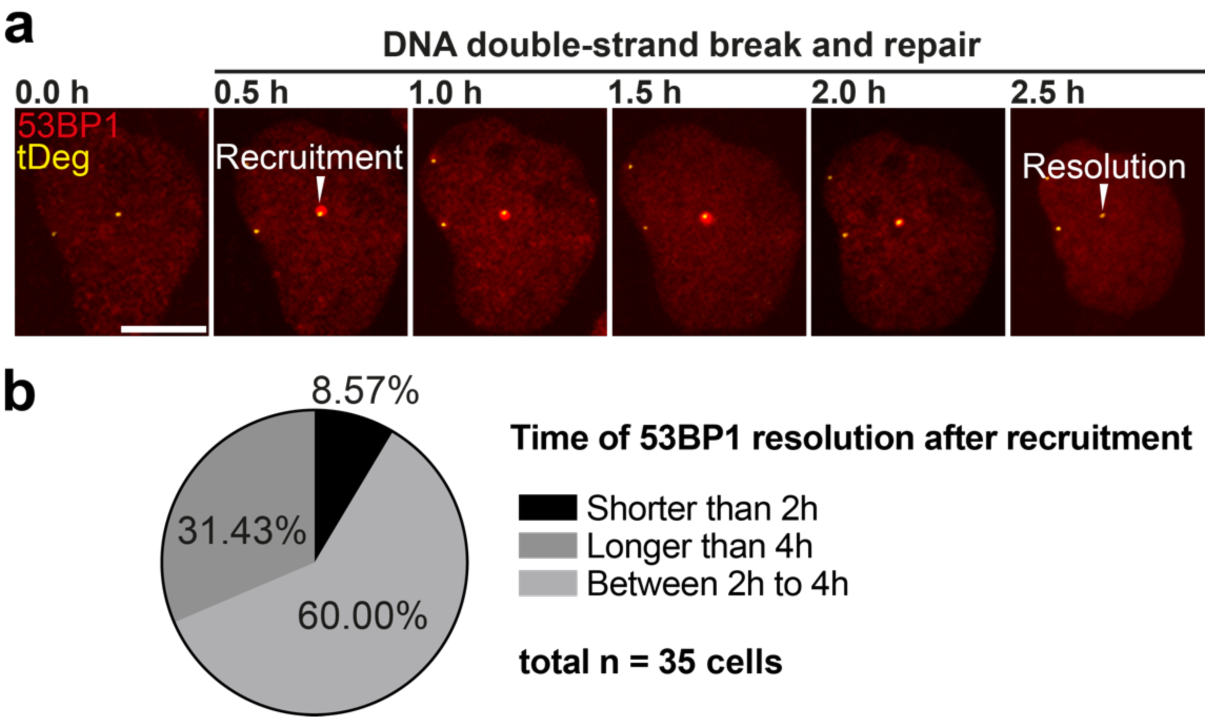

Supplementary Figure 16. The timing of repair after Cas9-induced DSBs.

a, The whole cell images of Fig. 6b. The corresponding 3D reconstruction was shown in Supplementary Movie 5. Scale bar, 5 µm.

b, Statistics of 53BP1 dwell times. We analyzed the repairing time after DSBs by observing the dwell time of 53BP1. Pie chart showing the percentages of different repair durations across sample cells (n = 35 cells). 8.57% of foci persisted for <2 h (black), 60.00% persisted for 2-4 h (light gray), and 31.43% persisted for >4 h (dark gray). The majority of alleles required 2 to 4 h for repairing after Cas9-induced DSBs, which is consistent with previous reports[12]. Source data are provided as a Source Data file.

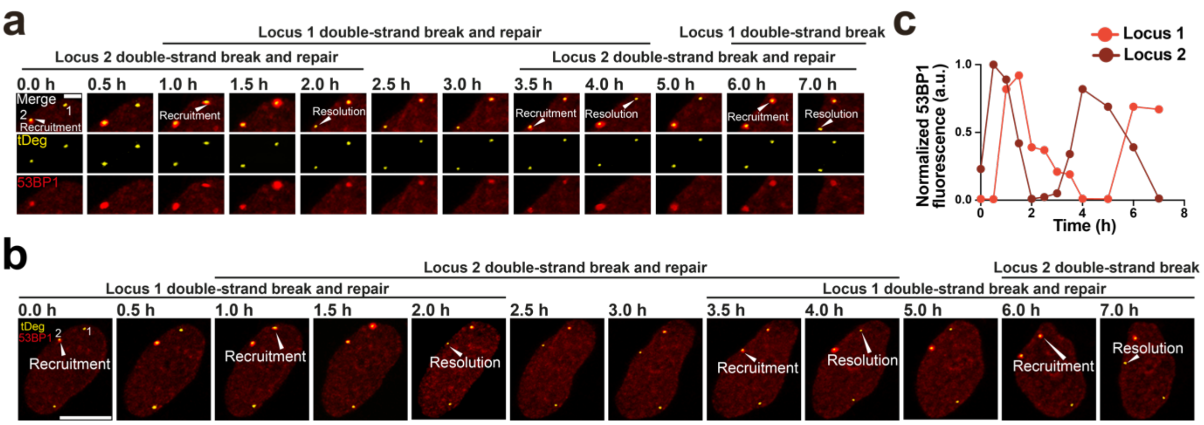

Supplementary Figure 17. fCRISPR enables to image the repeated cutting and repairing after Cas9-induced DSBs in Chromosome 3.

a, Representative images of DNA repeated cutting and repairing at *PPP1R2* locus. To distinguish the two Chromosome 3 loci, we labeled them as locus 1 and 2, respectively. These images showed that 53BP1-Apple were recruited at locus 1 at 0 h and dissociated at 2 h. Meanwhile, 53BP1-Apple were recruited at locus 2 at 1 h and dissociated at 4 h. 53BP1-Apple reporters were recruited to locus 1 again at 3.5 h and dissociated at 7 h. Similarly, 53BP1-Apple reporter were recruited to locus 2 again at 6 h. Images were acquired with the Olympus SpinSR-10 microscopy at 0.5 h time intervals. Z-stacks images were obtained at all time points. Image acquisition time, 7 h. The whole cells images and 3D reconstruction were shown in Supplementary Fig. 17b and Supplementary Movie 6. Scale bar, 5 μm.

b, The whole cell images of Supplementary Fig. 17a, showing repeated induction of DNA double-strand breaks and subsequent repair at a chromosome locus (white arrowheads). Maximum intensity projections of the Z-stacks are displayed. The corresponding 3D reconstruction was shown in Supplementary Movie 6. Scale bar, 5 μm.

c, Normalized fluorescence intensity of the 53BP1-Apple reporter during DNA repeated cutting and repairing at *PPP1R2* locus shown in Supplementary Fig. 17a. The normalized fluorescence of locus 1 (dark red) was gradually decreased after the initial increment, then gradually increased which represents the 53BP1 recruited to locus 1 again. The final decrement represents the 53BP1 resolution. The normalized fluorescence of locus 2 (orange) was shown a similar increase and decrease, which represents the 53BP1-Apple repeated recruitment and dissociation at locus 2. Data were analyzed by Fiji, and processed by GraphPad Prism 9. Source data are provided as a Source Data file.

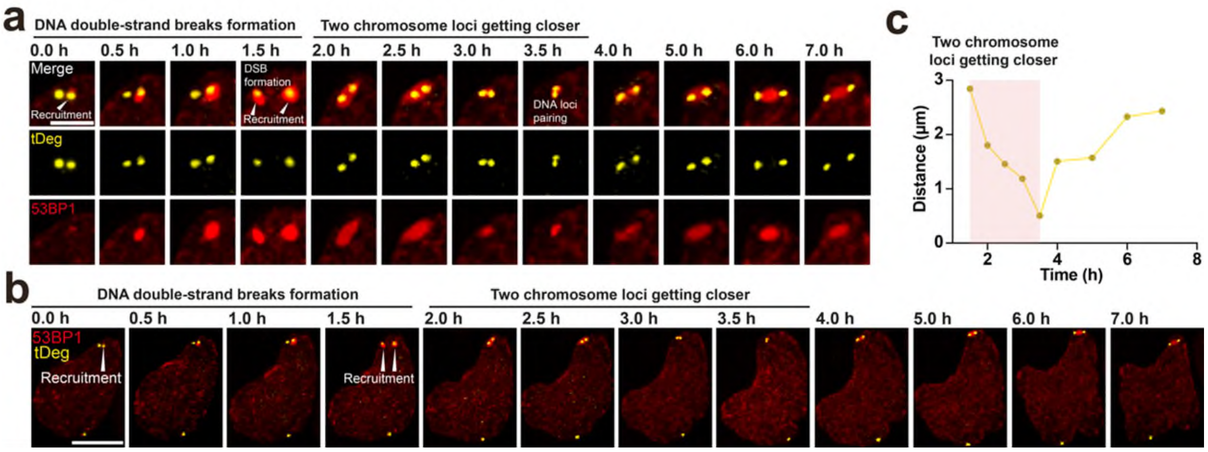

Supplementary Figure 18. Two chromosome loci gradually get closer after Cas9-induced DSBs in Chromosome 3.

a, Representative images showing 53BP1 foci fusion after 53BP1 was recruited to both loci sequentially (white arrowheads). Imaging showed that 53BP1 recruited two Chromosome 3 loci from 0 h to 1.5 h. Starting from 2 h point, the two loci were gradually closed at 3.5 h[11]. Then these two loci were gradually dissociated from 4 to 7 h. Scale bar, 5 µm.

b, The whole cell images of Supplementary Fig. 18a show the two chromosome 3 loci getting closer during repairing (white arrowheads), as well as the unrepaired gene locus at the bottom. The corresponding 3D reconstruction was shown in Supplementary Movie 7. Scale bar, 5 µm.

c, The distance of these two Chromosome 3 loci labeled by fCRISPR with YPet-tDeg reporter from 1 to 7 h. These two Chromosome 3 loci were gradually approached from 1 h to 3.5 h (light red region) shown in Supplementary Fig. 18a. Source data are provided as a Source Data file.

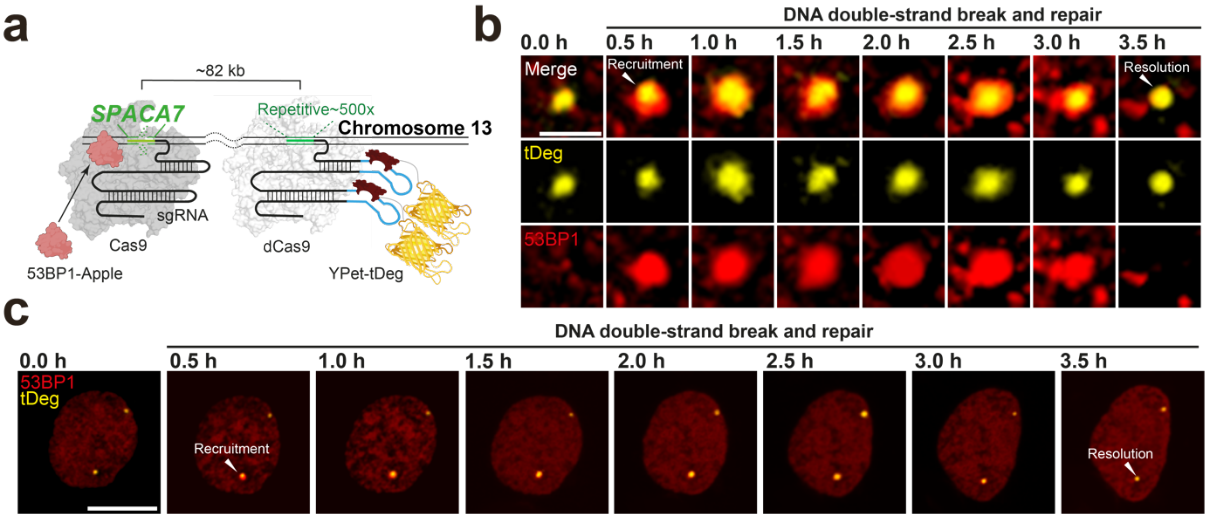

Supplementary Figure 19. Time-lapse imaging of *SPACA7* locus in Chromosome 13 DSBs and repair using fCRISPR.

a, Schematic of fCRISPR with editing CRISPR for tracking of DNA breaks and repairs of *SPACA7* locus in Chromosome 13 DSBs. Supplementary Fig. 19a was created with BioRender.com.

b, Representative images of DNA breaks and repairs during the recruitment and resolution of 53BP1at Chromosome 13 loci. Images were acquired with the Olympus SpinSR-10 microscopy at 0.5 h time intervals. Image acquisition time, 3.5 h. Scale bar, 5 μm.

c, The whole cell images of Supplementary Fig. 19b, showing the *SPACA7* locus breaks and repairs in the Chromosome 13 (white arrowheads). The corresponding 3D reconstruction was shown in Supplementary Movie 8. Scale bar, 5 μm.

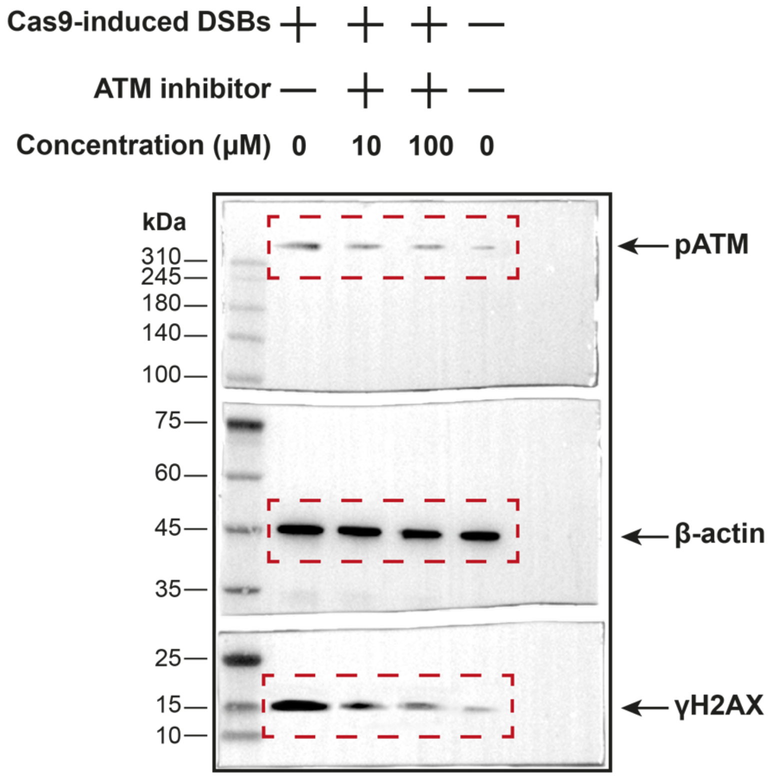

Supplementary Figure 20. The whole cropped blots in the WB experiment for Supplementary Figure14 i.

Supplementary Table 1. Plasmids used in each experiment.

Assay	Plasmids usage	Related to Figures
**Chromosome 3 targeted by fCRISPR**	0.2 µg dCas9-GFP + 0.5 µg Pepper-fused sgRNA targeting Chromosome 3 + 0.3 µg tdTomato-tDeg	Fig.1 b,c; Fig. 2a; Fig. 4a; Supplementary Fig. 3b; Supplementary Fig. 12a
**Colocalization between dCas9 and tDeg targeted low-copy genomic loci**	0.2 µg dCas9-GFP + 0.5 µg Pepper-fused sgRNA targeting Chromosome 9 with 30 copies + 0.3 µg tdTomato-tDeg; or 0.2 µg dCas9-GFP + 0.5 µg Pepper-fused sgRNA targeting Chromosome 3 with 25 copies + 0.3 µg tdTomato-tDeg; or 0.2 µg dCas9-GFP + 0.5 µg Pepper-fused sgRNA targeting Chromosome 9 with 17 copies+ 0.3 µg tdTomato-tDeg; 0.5 µg Pepper-fused sgRNA targeting Chromosome 13 with 14 copies + 0.3 µg tdTomato-tDeg	Fig. 2b; Supplementary Fig. 8a
**Colocalization between dCas9 and tDeg targeted different genomic loci**	0.2 µg dCas9-GFP + 0.5 µg Pepper-fused sgRNA targeting telomere + 0.3 µg tdTomato-tDeg; or 0.2 µg dCas9-GFP + 0.5 µg Pepper-fused sgRNA targeting Chromosome 3 + 0.3 µg tdTomato-tDeg; or 0.2 µg dCas9-GFP + 0.5 µg Pepper-fused sgRNA targeting Chromosome 13 + 0.3 µg tdTomato-tDeg; 0.5 µg Pepper-fused sgRNA targeting MUC4-I2 + 0.3 µg tdTomato-tDeg	Supplementary Fig. 7b
**tDeg with various fluorescent proteins**	0.2 µg dCas9-mCherry + 0.5 µg Pepper-fused sgRNA targeting Chromosome 3 + 0.3 µg mCerulean-tDeg; 0.2 µg dCas9-mCherry + 0.5 µg Pepper-fused sgRNA targeting Chromosome 3 + 0.3 µg mNeonGreen-tDeg; 0.2 µg dCas9-BFP + 0.5 µg Pepper-fused sgRNA targeting Chromosome 3 + 0.3 µg YPet-tDeg; 0.2 µg dCas9-GFP + 0.5 µg Pepper-fused sgRNA targeting Chromosome 3 + 0.3 µg tdTomato-Deg; 0.2 µg dCas9-GFP + 0.5 µg Pepper-fused sgRNA targeting Chromosome 3 + 0.3 µg iRFP670-tDeg	Fig. 3a; Supplementary Fig. 9
**Chromosome 3 and 13 dual-labeling (low copies)**	0.2 µg dCas9-BFP + 0.5 µg Broccoli-fused sgRNA targeting Chromosome 3 with 25 copies + 0.5 µg Pepper-fused sgRNA targeting Chromosome 13 with 14 copies + tdTomato-tDeg	Fig. 3c
**Chromosome 3 and 13 dual-labeling**	0.2 µg dCas9-BFP + 0.5 µg Broccoli-fused sgRNA targeting Chromosome 3 + 0.5 µg Pepper-fused sgRNA targeting Chromosome 13 + tdTomato-tDeg	Supplementary Fig. 10c
**DNA DSBs and repair in *PPP1R2***	For image DNA loci: 0.2 µg dCas9 + 0.5 µg Pepper-fused sgRNA targeting Chromosome 3 + 0.3 µg YPet-tDeg<br><br>For DNA double-strand break: 0.5 µg Cas9 with sgRNA targeting *PPP1R2*	Fig. 6b; Supplementary Fig. 14c,e; Supplementary Fig. 16a; Supplementary Fig. 17; Supplementary Fig. 18
**DNA DSBs and repair in *SPACA7***	For image DNA loci: 0.2 µg dCas9 + 0.5 µg Pepper-fused sgRNA targeting Chromosome 3 + 0.3 µg YPet-tDeg<br><br>For DNA double-strand break: 0.5 µg Cas9 with sgRNA targeting *SPACA7*	Supplementary Fig. 19
**Pepper: tDeg mechanism validation**	0.7 µg Tornado-Pepper + 0.3 µg tdTomato-tDeg	Supplementary Fig. 1
**MS2-MCP and fCRISPR comparison**	0.2 µg dCas9-BFP + 0.5 µg MS2-fused sgRNA targeting Chromosome 3 + 0.3 µg GFP-MCP	Supplementary Fig. 3b; Supplementary Fig. 12a
**dCas9-tdTomato and fCRISPR comparison**	0.2 µg dCas9-tdTomato + 0.8 µg sgRNA targeting Chromosome 3	Supplementary Fig. 4
**Broccoli-fused CRISPR**	0.2 µg dCas9 + 0.8 µg Broccoli fused sgRNA targeting Chromosome 3 with 25 copies, or 0.2 µg dCas9 + 0.8 µg Broccoli fused sgRNA targeting Chromosome 3 with 20 copies	Supplementary Fig. 11a,b
**DSBs validation**	0.5 µg Cas9 with sgRNA targeting *PPP1R2*	Supplementary Fig. 14a,g,i; Supplementary Fig. 15;
**fCRISPR for telomere length**	0.2 µg dCas9-GFP + 0.5 µg Pepper-fused sgRNA targeting telomere + 0.3 µg tdTomato-tDeg	Fig. 5; Supplementary Fig. 13

Supplementary Table 2. sgRNA and FISH probes sequences used in this study.

Pepper-fused sgRNA:
5' – NNNNNNNNNNNNNNNNNNNNGUUUGAGAGCUAGGCCGGCUCGUUGAGCUCAUUAGCUCCGAGCCGGCCUAGCAAGUUCAAAUAAGGCUAGUCCGUUAUCAACUUGGCCGGCUCGUUGAGCUCAUUAGCUCCGAGCCGGCCAAGUGGCACCGAGUCGGUGCUUUUUUU–3'

Broccoli-fused sgRNA:
5' – NNNNNNNNNNNNNNNNNNNNGUUUGAGAGCUAGGCCAGACGGTCGGGTCCAAATGAGACGGTCGGGTCCAGAAGTTCGCTTCTGTCGAGTAGAGTGTGGGCTCATTTGTCGAGTAGAGTGTGGGCTGGCCUAGCAAGUUCAAAUAAGGCUAGUCCGUUAUCAACUUGGCCAGACGGTCGGGTCCATCTGAGACGGTCGGGTCCAGTAGTTCGCTACTGTCGAGTAGAGTGTGGGCTCAGATGTCGAGTAGAGTGTGGGCTGGCCAAGUGGCACCGAGUCGGUGCUUUUUUU–3'

MS2-fused sgRNA:
5' – NNNNNNNNNNNNNNNNNNNNGUUUGAGAGCUAGGCCAACAUGAGGAUCACCCAUGUCUGCAGGGCCUAGCAAGUUCAAAUAAGGCUAGUCCGUUAUCAACUUGGCCAACAUGAGGAUCACCCAUGUCUGCAGGGCCAAGUGGCACCGAGUCGGUGCUUUUUUU–3'

The sequences of DNA FISH probes are:
Chromosome 3: Cy3/FITC - CTTCCTGTCACCGAC
Chromosome 13: Cy3/FITC - GACCATTCCTTCAGG

Supplementary Table 3. The spacer of sgRNA used in this study.

Spacer of sgRNA		Sequences information						Labeling and assay			
		Chromosome	Start	End	Spacer	PAM	Copies	fCRISPR		Broccoli-BI	
	1	Telomere	-	-	TAGGGTTAGGGTTAGGGTTA	GGG	-	Detectable	used		
	2	Centromere	-	-	GAATCTGCAAGTGGATATT	GGG	-	Detectable	used		
High copy number	3	Chromosome 3	198176561	198213923	GTGATATCACAG	TGG	~500	Detectable	used	Detectable	used
	4	Chromosome 13	111520792	111565328	GACCATTCCTTC	AGG	~500	Detectable	used		
	5	*MUC4*-I1	145956	149750	GAAGGTATGGGTGTGGAAGGTAT	TGG	~90	Detectable	used		
	6	Chromosome 3	76779382	76780192	CGGGTAGGGAGTAGGAGGTG	AGG	28	Detectable	used	Detectable	used
	7	Chromosome 3	197825777	197827851	CCACTGTCCACATCCTGCAG	CGG	27	Detectable	used	Detectable	used
	8	Chromosome 3	195505804	195515382	AAGTGTCGGTGACAGGAAGA	AGG	25	Detectable	used	Detectable	used; FISH
	9	Chromosome 3	196188307	196189591	GAGTTGAGTCTGTATTGTAG	GGG	20	Detectable	used; FISH	Detectable	used; FISH
	10	Chromosome 3	77339073	77345429	ATGGTTAAACGGGTAAGCTG	AGG	19	Detectable	used	Non-detectable	used
	11	Chromosome 3	197647881	197648915	CTACTGGTTTTCAGTAACTC	CGG	18	Detectable	used	Non-detectable	used
	12	Chromosome 9	140713060	140714036	CCGTAGAGAGGCCGACTGAGGGG	GGG	17	Detectable	used		
	13	Chromosome 9	320478	322273	CATCTCTGGTCACAGAACCTGGG	GGG	13	Non-detectable	used		
	14	Chromosome 9	140427595	140428594	ATTCTGGGAGTCCTCCCGCCTGG	TGG	8	Non-detectable	used		
Low copy number	15	Chromosome 13	114849103	114852585	ATCGACTCCCTTCCTCCTCG	TGG	25	Detectable	used		
	16	Chromosome 13	114885101	114886041	ATCGACGCCTGGCTGGAGAG	AGG	22	Detectable	used		
	17	Chromosome 13	114428290	114429450	ATCCAGGGGCGTGCACAGGGCGG	CGG	18	Detectable	used		
	18	Chromosome 13	77068741	77069631	GCTGAGGAGTGATCCCCAGC	AGG	14	Detectable	used; FISH		
	19	Chromosome 13	111149086	111149112	CACCAGCACAGCCTCAGTCA	AGG	10	Non-detectable	used		
	20	Chromosome 13	27623066	27623224	AAGTGGGAATGAGTTGGAGG	AGG	5	Non-detectable	used		
	21	Chromosome 19	269752	271115	TCACACGGACAC	NGG	20	Detectable	used		
	22	Chromosome 19	2347095	2350521	CACCTCTCTCTT	NGG	25	Detectable	used		
	23	Chromosome 19	5043100	5044468	TTTATCGGAGTG	NGG	28	Detectable	used		
	24	Chromosome 19	403972	405094	TCCTGCCCTGAG	NGG	30	Detectable	used		
	25	*PPP1R2*	-	-	GCTCCTCGTCGACATTCCCG	CGG	1	DSBs			
Other spacer	26	*SPACA7*	-	-	AATTCTCAGAAGGATCGCCA	TGG	1	DSBs			
	27	Scrambled element	-	-	TTCCGCGTTACATAACTTA	NGG	-	Background validation			

Supplementary References

1 Wu, J. *et al.* Live imaging of mRNA using RNA-stabilized fluorogenic proteins. *Nature Methods* **16**, 862-865, doi:10.1038/s41592-019-0531-7 (2019).

2 Nishimasu, H. *et al.* Crystal Structure of Cas9 in Complex with Guide RNA and Target DNA. *Cell* **156**, 935-949, doi:10.1016/j.cell.2014.02.001 (2014).

3 Konermann, S. *et al.* Genome-scale transcriptional activation by an engineered CRISPR-Cas9 complex. *Nature* **517**, 583-588, doi:10.1038/nature14136 (2015).

4 Ma, H. *et al.* Multiplexed labeling of genomic loci with dCas9 and engineered sgRNAs using CRISPRainbow. *Nature Biotechnology* **34**, 528-530, doi:10.1038/nbt.3526 (2016).

5 Ma, H. *et al.* CRISPR-Sirius: RNA scaffolds for signal amplification in genome imaging. *Nat. Meth.* **15**, 928-931, doi:10.1038/s41592-018-0174-0 (2018).

6 Ren, L. *et al.* Potential biomarkers of DNA replication stress in cancer. *Oncotarget* **8** (2017).

7 Qin, P. *et al.* Live cell imaging of low- and non-repetitive chromosome loci using CRISPR-Cas9. *Nature Communications* **8**, 14725, doi:10.1038/ncomms14725 (2017).

8 Chen, B. *et al.* Dynamic Imaging of Genomic Loci in Living Human Cells by an Optimized CRISPR/Cas System. *Cell* **155**, 1479-1491, doi:https://doi.org/10.1016/j.cell.2013.12.001 (2013).

9 Li, X., Kim, H., Litke, J. L., Wu, J. & Jaffrey, S. R. Fluorophore-Promoted RNA Folding and Photostability Enables Imaging of Single Broccoli-Tagged mRNAs in Live Mammalian Cells. *Angew. Chem., Int. Ed.* **59**, 4511-4518, doi:10.1002/anie.201914576 (2020).

10 Chen, B. *et al.* Dynamic imaging of genomic loci in living human cells by an optimized CRISPR/Cas system. *Cell* **155**, 1479-1491, doi:10.1016/j.cell.2013.12.001 (2013).

11 Wang, H. *et al.* CRISPR-mediated live imaging of genome editing and transcription. *Science* **365**, 1301-1305, doi:doi:10.1126/science.aax7852 (2019).

12 Liu, Y. *et al.* Very fast CRISPR on demand. *Science* **368**, 1265-1269, doi:10.1126/science.aay8204 (2020).

13 Diehl, M. C., Elmore, L. W. & Holt, S. E. in *Telomeres and Telomerase in Cancer* (ed Keiko Hiyama) 87-125 (Humana Press, 2009).

14 Chwastek, J., Jantas, D. & Lasoń, W. The ATM kinase inhibitor KU-55933 provides neuroprotection against hydrogen peroxide-induced cell damage via a γH2AX/p-p53/caspase-3-independent mechanism: Inhibition of calpain and cathepsin D. *The International Journal of Biochemistry & Cell Biology* **87**, 38-53, doi:https://doi.org/10.1016/j.biocel.2017.03.015 (2017).

15 Celeste, A. *et al.* Histone H2AX phosphorylation is dispensable for the initial recognition of DNA breaks. *Nature Cell Biology* **5**, 675-679, doi:10.1038/ncb1004 (2003).

16 Kinner, A., Wu, W., Staudt, C. & Iliakis, G. Gamma-H2AX in recognition and signaling of DNA double-strand breaks in the context of chromatin. *Nucleic Acids Res* **36**, 5678-5694, doi:10.1093/nar/gkn550 (2008).

17 Redon, C. E., Dickey, J. S., Bonner, W. M. & Sedelnikova, O. A. γ-H2AX as a biomarker of DNA damage induced by ionizing radiation in human peripheral blood lymphocytes and artificial skin. *Advances in Space Research* **43**, 1171-1178, doi:https://doi.org/10.1016/j.asr.2008.10.011 (2009).
