# Supplementary material for: Fluorogenic CRISPR for genomic DNA imaging
Source: Nat Commun. 2024 Jan 31;15:934. doi: 10.1038/s41467-024-45163-9 (PMC10831063; doi:10.1038/s41467-024-45163-9)
Supplement: Supplementary file 3 — Description of Additional Supplementary Files [file 41467_2024_45163_MOESM3_ESM.pdf]

Description of Additional Supplementary Files:

Supplementary Movie 1: To track the Chromosome 3 dynamics, we observed three Chromosome 3 loci are labeled by fCRISPR with tdTomato-tDeg reporter (red). Images were taken with a confocal microscope (Olympus SpinSR10), spaced by 300 ms with a total of 34 shots. Display rate 6 frames/s.

Supplementary Movie 2: To compare the chromosome dynamics using different CRISPR-based approaches, we first use fCRISPR to track Chromosome 3. Three Chromosome 3 loci are labeled by fCRISPR with tdTomato-tDeg reporter (red). Images were taken with a confocal microscope (Olympus SpinSR10), spaced by 117 ms with a total of 128 shots. Display rate 20 frames/s.

Supplementary Movie 3: We next used the conventional CRISPR with dCas9-GFP reporter to track Chromosome 3. Three Chromosome 3 loci are labeled by fCRISPR with dCas9-GFP reporter (green). Images were taken with a confocal microscope (Olympus SpinSR10), spaced by 117 ms with a total of 128 shots. Display rate 20 frames/s.

Supplementary Movie 4: We finally used MS2-fused CRISPR with MCP-GFP reporter to track Chromosome 3. Three Chromosome 3 loci are labeled by MS2-fused CRISPR with MCP-GFP reporter (green). Images were taken with a confocal microscope (Olympus SpinSR10), spaced by 117 ms with a total of 128 shots. Display rate 20 frames/s.

Supplementary Movie 5: The DNA DSBs at PPP1R2 locus and repairs process were observed in 2.5 h with 0.5 h intervals. Z-slides were obtained with ~2 µm space. Whole-cell zstack images were re-construction using Imaris x64 (ver. 9.0.1) with the 3D viewer (convolution) and animation functions to create the movie in chronological order as shown. The subtitle of each time point was added in the movie to represent the DSBs repairing procedure. The movies of each time point were finally interrogated in a single file.

Supplementary Movie 6: The repeated repairing after DNA DSBs at PPP1R2 locus was observed in 7 h with 0.5 h intervals. The images with each time point were shown in Supplementary Fig. 17.

Supplementary Movie 7: The initially separate Chromosome 3 loci then draw near during repairing after DNA DSBs at PPP1R2 locus in Chromosome 3 were observed in 7 h with 0.5 h intervals. The truncated images with each time point were shown in Supplementary Fig. 18a, and the whole cell images were shown in Supplementary Fig. 18b. The Chromosome 3 puncta of 1 and 2 were labeled in the movie. The process of 3D reconstruction and the production of the movie as mentioned.

Supplementary Movie 8: The DNA DSBs at SPACA7 locus and repairs process were observed in 3.5 h with 0.5 h intervals. The truncated images with each time point were shown in Supplementary Fig. 19b, and the whole cell images were shown in Supplementary Fig. 19c. The process of 3D reconstruction and the production of the movie as mentioned.
