# Supplementary material for: Fluorogenic CRISPR for genomic DNA imaging
Source: Nat Commun. 2024 Jan 31;15:934. doi: 10.1038/s41467-024-45163-9 (PMC10831063; doi:10.1038/s41467-024-45163-9)
Supplement: Supplementary file 12 — Reporting Summary [file 41467_2024_45163_MOESM12_ESM.pdf]

nature portfolio

Corresponding author(s): Xing Li

Last updated by author(s): Nov 16, 2023

Reporting Summary

Nature Portfolio wishes to improve the reproducibility of the work that we publish. This form provides structure for consistency and transparency in reporting. For further information on Nature Portfolio policies, see our Editorial Policies and the Editorial Policy Checklist.

Statistics

For all statistical analyses, confirm that the following items are present in the figure legend, table legend, main text, or Methods section.

n/a	Confirmed	
☐	☒	The exact sample size (n) for each experimental group/condition, given as a discrete number and unit of measurement
☐	☒	A statement on whether measurements were taken from distinct samples or whether the same sample was measured repeatedly
☐	☒	The statistical test(s) used AND whether they are one- or two-sided<br>*Only common tests should be described solely by name; describe more complex techniques in the Methods section.*
☒	☐	A description of all covariates tested
☒	☐	A description of any assumptions or corrections, such as tests of normality and adjustment for multiple comparisons
☐	☒	A full description of the statistical parameters including central tendency (e.g. means) or other basic estimates (e.g. regression coefficient) AND variation (e.g. standard deviation) or associated estimates of uncertainty (e.g. confidence intervals)
☐	☒	For null hypothesis testing, the test statistic (e.g. F, t, r) with confidence intervals, effect sizes, degrees of freedom and P value noted<br>*Give P values as exact values whenever suitable.*
☒	☐	For Bayesian analysis, information on the choice of priors and Markov chain Monte Carlo settings
☒	☐	For hierarchical and complex designs, identification of the appropriate level for tests and full reporting of outcomes
☒	☐	Estimates of effect sizes (e.g. Cohen's d, Pearson's r), indicating how they were calculated

Our web collection on statistics for biologists contains articles on many of the points above.

Software and code

Policy information about availability of computer code

Data collection	OLYMPUS cellSens Dimension (Build 21199) RELEASE X64 was used for collecting imaging data. Bio-Rad CFX96 system was used to collect the qPCR data. Tanon 5200 Chemiluminescent Imaging System was used to collect the western blots data. BD FACSDiva™ Software was used to collect the data for cell sorting.
Data analysis	FIJI 2.9.0 was used for imaging data processing. OriginPro 9.65, Excel 16.69.1, GraphPad Prism 9.4.1 were used for generating plots and bar graphs. BioRad CFX manager software was used for qPCR data analysis. Imaris x64 (ver. 9.0.1) was used to reconstruct Z-stack images.

For manuscripts utilizing custom algorithms or software that are central to the research but not yet described in published literature, software must be made available to editors and reviewers. We strongly encourage code deposition in a community repository (e.g. GitHub). See the Nature Portfolio guidelines for submitting code & software for further information.

Data

Policy information about availability of data

All manuscripts must include a data availability statement. This statement should provide the following information, where applicable:

- Accession codes, unique identifiers, or web links for publicly available datasets
- A description of any restrictions on data availability
- For clinical datasets or third party data, please ensure that the statement adheres to our policy

All other data supporting the findings of this study are available within the paper and the associated supplementary files.

Research involving human participants, their data, or biological material

Policy information about studies with human participants or human data. See also policy information about sex, gender (identity/presentation), and sexual orientation and race, ethnicity and racism.

Reporting on sex and gender	No human or animal research was performed.
Reporting on race, ethnicity, or other socially relevant groupings	No human or animal research was performed.
Population characteristics	No human or animal research was performed.
Recruitment	No human or animal research was performed.
Ethics oversight	No human or animal research was performed.

Note that full information on the approval of the study protocol must also be provided in the manuscript.

Field-specific reporting

Please select the one below that is the best fit for your research. If you are not sure, read the appropriate sections before making your selection.

- [x] Life sciences
- [ ] Behavioural & social sciences
- [ ] Ecological, evolutionary & environmental sciences

For a reference copy of the document with all sections, see nature.com/documents/nr-reporting-summary-flat.pdf

Life sciences study design

All studies must disclose on these points even when the disclosure is negative.

Sample size	No statistical methods were used to predetermine the sample size. Sample sizes were chosen due to being able to show reproducibility and statistical significance. In detail, the sample size (n) of each experiment is provided in the figure legends in the main manuscript and supplementary information file. For characterization of the data in live cells, final data sets are comprised of at least triplicate independent cell cultures. These sizes have previously been shown as sufficiently powered to determine statistical differences in the mean values of our investigated parameters.
Data exclusions	No data were excluded from the analyses.
Replication	We employed a two-tailed equal-variance Student's t-test to compute the p values, and a minimum of three trained independent investigators conducted cell counting in a blinded manner.
Randomization	Cells were randomly assigned into control or experimental groups. Cell images were randomly assigned to investigators for counting.
Blinding	Blinding was performed, as studies were potentially deemed to be influenced by human interpretation. For instance, we performed blinding in truncated 53BP1-Apple acts as DNA double-strand breaks sensors validation.

Reporting for specific materials, systems and methods

We require information from authors about some types of materials, experimental systems and methods used in many studies. Here, indicate whether each material, system or method listed is relevant to your study. If you are not sure if a list item applies to your research, read the appropriate section before selecting a response.

Materials & experimental systems

n/a	Involved in the study	
	x	Antibodies
	x	Eukaryotic cell lines
x		Palaeontology and archaeology
x		Animals and other organisms
x		Clinical data
x		Dual use research of concern
x		Plants

Methods

n/a	Involved in the study	
x		ChIP-seq
	x	Flow cytometry
x		MRI-based neuroimaging

Antibodies

Antibodies used	Primary antibodies for western blotting:

	-Phospho-ATM (Ser1981) (D6H9) (Cell Signaling Technologies, Rabbit mAb, cat#5883)<br>-Phospho-Histone H2A.X (Ser139) (20E3) (Cell Signaling Technologies, Rabbit mAb, cat#9718)<br>-β-Actin (13E5) (Cell Signaling Technologies, Rabbit mAb, cat#4970)<br>All at 1:1000 working dilution<br><br>Secondary antibodies for western blotting:<br>-Anti-rabbit IgG, HRP-linked (Cell Signaling Technologies, cat#7074)<br>At 1:10000 working dilution<br><br>Primary antibodies for immunofluorescence:<br>--Phospho-Histone H2A.X (Ser139) (20E3) (Cell Signaling Technologies, Rabbit mAb, cat#9718)<br>At 1:200 working dilution<br><br>Secondary antibodies for immunofluorescence:<br>-Anti-rabbit IgG (H+L), Alexa Fluor 488 conjugated (Cell Signaling Technologies, cat#4412)<br>At 1:500 working dilution
Validation	-Phospho-ATM (Ser1981) (D6H9) (Cell Signaling Technologies, Rabbit mAb, cat#5883): The antibody guarantee covers the use of the antibody for WB applications. The antibody has been referenced in 220 publications. https://www.cellsignal.com/products/primary-antibodies/phospho-atm-ser1981-d6h9-rabbit-mab/5883<br><br>-Phospho-Histone H2A.X (Ser139) (20E3) (Cell Signaling Technologies, Rabbit mAb, cat#9718): The antibody guarantee covers the use of the antibody for WB and immunofluorescence applications. The antibody has been referenced in 2071 publications.<br><br>-β-Actin (13E5) (Cell Signaling Technologies, Rabbit mAb, cat#5636): The antibody guarantee covers the use of the antibody for WB applications. The antibody has been referenced in 6477 publications.<br><br>-Phospho-ATM (Ser1981) (10H11.E12) (Cell Signaling Technologies, Mouse mAb, cat#4526): The antibody guarantee covers the use of the antibody for WB applications. The antibody has been referenced in 229 publications. https://www.cellsignal.com/products/primary-antibodies/phospho-atm-ser1981-10h11-e12-mouse-mab/4526

Eukaryotic cell lines

Policy information about cell lines and Sex and Gender in Research

Cell line source(s)	U2OS (ATCC-HTB-96), HeLa (ATCC-CCL-2), Huh7 (ATCC-CCL-185), LO2 (ATCC-HL-7702), HEK293T (ATCC-CRL-11268), RPE (ATCC-CRL-2302), and UMUC3 (ATCC-CRL-1749) cell lines were used in this study.
Authentication	No cell lines were authenticated.
Mycoplasma contamination	All cell lines have been tested negative for mycoplasma contamination by PCR methods.
Commonly misidentified lines (See ICLAC register)	None of the commonly misidentified lines have been used in this study.

Plants

Seed stocks	No plants were involved in this study.
Novel plant genotypes	No plants were involved in this study.
Authentication	No plants were involved in this study.

Flow Cytometry

Plots

Confirm that:

- [x] The axis labels state the marker and fluorochrome used (e.g. CD4-FITC).
- [ ] The axis scales are clearly visible. Include numbers along axes only for bottom left plot of group (a 'group' is an analysis of identical markers).
- [ ] All plots are contour plots with outliers or pseudocolor plots.
- [x] A numerical value for number of cells or percentage (with statistics) is provided.

Methodology

Sample preparation	Adherent cell lines were harvested from culture using trypsin.
Instrument	The FACSAria III with 561 nm excitation from the Institute of Genetics and Developmental Biology in the Chinese Academy of Science was used for this experiment.
Software	BD FACSDiva™ Software was used for cell sorting to collect low-expressing 53BP1-Apple U2OS cell lines.
Cell population abundance	Of the surviving single-sorted U2OS cells transduced to express 53BP1-Apple, and around 30% were low-expressing 53BP1-Apple, then collected.
Gating strategy	Cells were initially gated to FSC-A and SSC-A according to the observed singlets and doublets, selecting only singlets. Subsequently, the majority population showing a relatively lower 53BP1-Apple signal was selected for gating.

- [x] Tick this box to confirm that a figure exemplifying the gating strategy is provided in the Supplementary Information.
